# Development of non-viral vehicles for targeted gene transfer into microglia via the integrin receptor CD11b

**DOI:** 10.3389/fnmol.2014.00079

**Published:** 2014-10-09

**Authors:** Markus Smolny, Mary-Louise Rogers, Anthony Shafton, Robert A. Rush, Martin J. Stebbing

**Affiliations:** ^1^School of Medical Sciences and Health Innovations Research Institute, Royal Melbourne Institute of Technology UniversityBundoora, VIC, Australia; ^2^Department of Human Physiology, Centre for Neuroscience, Flinders UniversityAdelaide, SA, Australia; ^3^The Florey Institute of Neuroscience and Mental Health, The University of MelbourneParkville, VIC, Australia

**Keywords:** microglia, CD11b, OX42, non-viral vectors, polyethyleneimine (PEI), phagocytosis, respiratory burst

## Abstract

Microglial activation is a central event in neurodegeneration. Novel technologies are sought for that specifically manipulate microglial function in order to delineate their role in onset and progression of neuropathologies. We investigated for the first time whether non-viral gene delivery based on polyethyleneglycol–polyethyleneimine conjugated to the monoclonal anti-CD11b antibody OX42 (“OX42-immunogene”) could be used to specifically target microglia. We first conducted immunofluorescence studies with the OX42 antibody and identified its microglial integrin receptor CD11b as a potential target for receptor-mediated gene transfer based on its cellular specificity in mixed glia culture and *in vivo* and found that the OX42 antibody is rapidly internalized and trafficked to acidic organelles in absence of activation of the respiratory burst. We then performed transfection experiments with the OX42-immunogene *in vitro* and in rat brain showing that the OX42-immunogene although internalized was degraded intracellularly and did not cause substantial gene expression in microglia. Investigation of specific barriers to microglial gene transfer revealed that aggregated OX42-immunogene polyplexes stimulated the respiratory burst that likely involved Fcγ-receptors. Transfections in the presence of the endosomolytic agent chloroquine improved transfection efficiency indicating that endosomal escape may be limited. This study identifies CD11b as an entry point for antibody-mediated gene transfer into microglia and takes important steps toward the further development of OX42-immunogenes.

## INTRODUCTION

Microglia are the primary immune cells of the central nervous system (CNS) and exert many of their important functions through changes in morphology and gene expression termed “microglial activation” (reviewed in Ransohoff and Cardona, 2010; Schafer et al., 2012). It is unclear if activated microglia are neuroprotective, neurotoxic or neuromodulatory in neuropathology (Saijo and Glass, 2011; Aguzzi et al., 2013). Thus, methods are being sought for that allows specific manipulation of microglial function in order to gain more insight into their role.

Viruses have been used to manipulate gene expression in microglial cells. Nevertheless, the development of a virus-based transgene carrier specifically targeting microglia has been proven difficult, because most viruses display broad tropism and therefore require extensive modification to increase specificity (Burke et al., 2002; Cucchiarini et al., 2003; Pfrieger and Slezak, 2012). Further, the application of viral vectors *in vivo* was shown to bear risk of insertional mutagenesis. This was found especially with lentiviral vectors (Burke et al., 2002; Bokhoven et al., 2009) that are often used to transfect microglia and other glial cells *in vitro* and *in vivo* (Wrzesinski et al., 2000; Balcaitis et al., 2005; Meunier et al., 2007, 2008; McCoy et al., 2008; Dominguez et al., 2010; Lee et al., 2011; Jiang et al., 2012; Kim et al., 2012; Liu et al., 2013; Maiorino et al., 2013) and thus limits their use for other purposes than basic research such as gene therapy.

Non-viral vehicles have emerged as an alternative for gene delivery with advantages such as ability to target specific populations of cells and low immunotoxicity compared to viruses (reviewed in Lv et al., 2006 and Raety et al., 2008). The cationic polymer polyethyleneimine (PEI) has been frequently used to bind and condense plasmid DNA (pDNA), to protect it from degradation and facilitate endosomal escape (Akinc et al., 2005; Neu et al., 2005; Yue et al., 2011). Further, modification of PEI with polyethyleneglycol (PEG) was demonstrated to add stability to PEI complexes by decreasing aggregation (Tang et al., 2003; Mishra et al., 2004; Millili et al., 2010), reducing PEI-mediated toxicity (reviewed in Lungwitz et al., 2005), diminishing non-specific interaction of positively charged PEI with negatively charged proteoglycans on off-target cells (Ogris et al., 2001) and improving *in vivo* gene delivery (Germershaus et al., 2006; Duan et al., 2010).

Non-viral bioconjugates based on PEI–PEG and chemically linked to monoclonal antibodies for receptor targeting (herein referred to as “immunogenes”) may be a promising tool for specific modulation of microglial function. Antibodies may confer increased specificity compared to other ligands such as polysaccharides (Aouadi et al., 2009) and mannose receptor ligands (Ferkol et al., 1996, 1998; Kawakami et al., 2000; Markovic et al., 2009) which bind to receptors that are more ubiquitously expressed. Such antibody-based vehicles have been successfully applied previously to deliver genes to several cell lines *in vitro* (Kircheis et al., 1997) and motor neurons (Barati et al., 2006) and cholinergic basal forebrain neurons (Berhanu and Rush, 2008) *in vivo*.

Antibodies to the integrin receptor CD11b, also known as complement receptor 3 (CR3), have the potential to be used for targeting immunogenes to microglia. The CD11b receptor is involved in the immune response of microglia and macrophages of the CNS and the peripheral immune system, respectively (Akiyama and McGeer, 1990; Berton and Lowell, 1999; Milner and Campbell, 2002). CD11b expression is up-regulated in activated microglia in a variety of neuropathological conditions, for instance in the hypothalamic paraventricular nucleus (PVN) following myocardial infarction (Rana et al., 2010). While immune receptors may trigger strong pro-inflammatory immune responses such as reactive oxygen species (ROS) production and the respiratory burst (Nimmerjahn and Ravetch, 2008) which are unwanted side-effects of gene transfer, CD11b is interestingly involved in both, pro-inflammatory (Block et al., 2007; Pei et al., 2007; Zhang et al., 2007; Hu et al., 2008; Davalos et al., 2012) and anti-inflammatory (Griffiths et al., 2009; Amarilyo et al., 2010; Ricklin et al., 2010) immune functions in microglia that seem to depend on the receptor binding site (Větvčka et al., 1996; Xia et al., 1999; Ross, 2000) and interactions with other co-receptors such as Fcγ-receptors (Huang et al., 2011). More importantly, parasites and bacteria utilize CD11b to infect host cells and to avoid intracellular degradation (Mosser and Edelson, 1987; Hajishengallis and Lambris, 2011) indicating that CD11b may be also an entry point for antibody-mediated delivery of transgenes into microglia. The monoclonal antibody OX42 targets CD11b (Robinson et al., 1986) and is suggested to bind at or close to a site of CD11b related to its anti-inflammatory properties (Klegeris and McGeer, 1994; Sohn et al., 2003). Thus, OX42 may be suitable as a targeting ligand for microglial-specific gene delivery combined with the ability of PEI–PEG to bind, protect and transfer DNA into cells.

We thus propose to determine the specificity and suitability of the monoclonal antibody OX42 as a targeting agent for immunogenes for microglia and whether a targeting bioconjugate (“OX42-immunoporter”) could be used for specific microglial transfection. Immunofluorescence studies with labeled OX42 antibody were conducted in cultured glia cells as well as *in vivo* to determine the specificity of CD11b for microglia, the ability of CD11b to internalize the antibody and its intracellular localization. Subsequent transfection experiments with the targeting bioconjugate were then conducted *in vitro* and *in vivo* utilizing immunohistochemistry (IHC) to determine successful microglial transfection. The activation of the respiratory burst by aggregated polyplexes as measured by dynamic light scattering (DLS) and an assay for ROS as well as limited endosomal escape as determined by transfections in presence of the endosomolytic agent chloroquine was then investigated to identify potential microglia-specific barriers to non-viral gene transfer. This study is the first to describe CD11b as a target on microglia for receptor-mediated gene transfer and to identify microglia-specific barriers that will lead to further development of targeting non-viral gene vehicles for microglial gene transfer.

## MATERIALS AND METHODS

### ANIMAL ETHICS

All procedures performed on animals were approved by the RMIT University Institutional Animal Experimentation Ethics Committee or the Alfred Medical Research and Education Precinct Animal Ethics Committee and conformed to the National Health and Medical Research Council of Australia code of practice for the care and use of animals for scientific purposes.

### EXPERIMENTAL OVERVIEW

*In vitro* experiments for specificity and internalization of the OX42 antibody as well as transfections were performed in mixed cultures and isolated microglia obtained from 1 to 3 day old Sprague-Dawley rat brains as outlined in Section “Primary Cell Culture.” A total of five brains of 9–10 weeks old male Sprague-Dawley rats (300–350 *g*) were used to test specificity of CD11b for microglia and for *in vivo* transfections. Studies on barriers to microglial transfections *in vitro* utilized mixed glia cultures and isolated microglia.

### PRIMARY CELL CULTURE

Mixed glia cultures were prepared from neonatal Sprague-Dawley rat brains (days 1–3) based on the method developed by Nakajima et al. (1989). Briefly, a cell suspension was obtained by combination of mechanical and enzymatical (0.16% trypsin/0.01% deoxyribonuclease I, Sigma) dissociation of brain tissue. Mixed glia cultures were maintained in Dulbecco’s Modified Eagle Medium with high glucose (DMEM, Gibco^TM^) supplemented with 10% fetal bovine serum (FBS, Bovogen) and 2% penicillin-streptomycin (Gibco^TM^) in poly-D-lysine (PDL, Sigma)-coated tissue culture flask (25 cm^2^, TPP) exchanging half of the cell culture medium twice a week. Microglial cells appeared on top of an astrocytic layer after a few days and were available in sufficient amounts for isolation after 8–12 days. For immunofluorescence studies and transfections that involved mixed glia cultures, mixed cultures were prepared as above, but plated on PDL-coated coverslips in 24-well plates. Mixed cultures were used for experiments 3–4 days thereafter.

Microglial cells were isolated from mixed cultures by shaking the cell culture flasks at 37°C for 60 min (120 rpm). Dependent on the experiment, isolated microglia (>98% pure, determined by CD11b-immunoreactivity with OX42 antibody, data not shown) were either plated on PDL-coated coverslips (2 × 10^4^ cells/well), onto PDL-coated fluorodishes (World Precision Instruments, 5 × 10^4^ cells/dish) or in PDL-coated black 96-well plates (Greiner, 1 × 10^4^ cells/well).

### ANTIBODY PURIFICATION AND LABELING

OX42 and X63 antibody secreting hybridoma cell lines were grown in RPMI-1640 GlutaMax medium supplemented with 10% FBS, 1× penicillin-streptomycin-glutamine and 1× hypoxanthine-thymidine (Gibco^TM^). The antibody X63 has no known antigen and was used as non-specific control IgG. Monoclonal antibodies were purified from supernatant using protein G (Millipore) according to the manufacturer’s instructions.

Antibodies were labeled according to manufacturer’s instructions with either *N*-hydroxysuccinimide (NHS)-activated fluorescein or Alexa 488 sulfodichlorophenol (SDP) ester (Molecular Probes®;). Tagged antibodies were purified on PD10 desalting columns (GE Healthcare). The fluorophore to protein (F/P) ratios obtained were F/P = 9 for OX42-FITC, 5 for OX42-Alexa488, and 4 for X63-Alexa488.

### IMMUNOCYTOCHEMISTRY AND IMMUNOHISTOCHEMISTRY

Double-labeling experiments in mixed glia culture and in brain slices were performed to either determine specificity of OX42 antibody for microglia or assess transfections *in vitro* and *in vivo*. Cultures were fixed [4% paraformaldehyde (PFA), Merck] and then blocked for 30 min (10% normal horse serum, 0.1% Triton-X100, Sigma), while brain sections were blocked in 10% normal horse serum containing 0.5% Triton-X100 (1 h, room temperature). Antibodies against cell markers (**Table 1**) were used to detect astrocytes [anti-glial fibrillary acidic protein (GFAP)] and microglia [ionized calcium binding adapter molecule 1, (Iba1); or CD11b]. Green fluorescent protein (EGFP) expression in transfected cells was confirmed with a monoclonal mouse anti-EGFP antibody. Labeled secondary antibodies were applied to visualize primary antibody binding.

**Table 1:** Antibodies and reagents used for immunostaining.

Target	Antibody	Tag	Host	Use	c/DF	Source
CD11b	OX42	–	Mouse	ICC	1.25 μg/mL	RMIT
Iba1	α-Iba1	–	Goat	ICC	400	Abcam
				IHC	100	
GFAP	α-GFAP	–	Rabbit	ICC/IHC	150	Life Technologies
EGFP	α-GFP	–	Mouse	ICC	400	Roche
				IHC	200	
Mouse IgG	α-mouse	Alexa488	Donkey	ICC	400	Life Technologies
Mouse IgG	α-mouse	Biotin	Horse	ICC	400	Vector Labs
Goat IgG	α-goat	Biotin	Horse	ICC	400	Vector Labs
Rabbit IgG	α-rabbit	Alexa594	Donkey	ICC/IHC	400	Life Technologies
Biotin	Extravidin	Cy3	–	ICC/IHC	600	Sigma

*c, concentration; DF, dilution factor; ICC, immunocytochemistry; IHC, immunohistochemistry; GFAP, glial fibrillary acidic protein; Iba1, ionized calcium binding adapter molecule 1.*

All antibody incubations in mixed culture were performed for 1 h at room temperature. For IHC, primary antibodies were incubated for 3 days at 2–8°C in a humidifying chamber and secondary antibodies applied thereafter for 2 h at room temperature followed by 1 h incubation with extravidin-Cy3 conjugate. Cell nuclei were counter-stained with Hoechst dye (1:500, 10–25 min).

Specific staining by primary antibodies was demonstrated *in vitro* by following the same immunostaining methods but omitting the primary antibodies (data not shown). Each condition was run on duplicate coverslips in each experiment and at least three experiments were performed.

### CELLULAR DISTRIBUTION OF CD11b *IN VIVO*

Male Sprague-Dawley rats (300–350 *g*) were stereotactically injected under isoflurane anesthesia with 250–300 nL of Alexa 488-labeled OX42 antibody (right side) or Alexa 488-labeled X63 antibody (left side) into the PVN of the hypothalamus of the same animal (0.6 mm lateral from midline, 8.0 mm depth, –2.1 mm posterior from Bregma). Animals were kept anesthetized and perfused (4% PFA, Merck) 3 h after injections before brains were collected and post-fixed for 4 h. After 3–4 days of storage in 30% sucrose, 30 μm brain sections were cut on a cryostat, placed on gelatin-coated microscope slides and IHC performed. Two animals were injected and localization of OX42 antibody was assessed qualitatively by IHC.

### INTERNALIZATION OF OX42 ANTIBODY

Isolated microglial cells on coverslips were incubated with 2 μg/mL FITC-tagged OX42 antibody at 37°C, fixed after 5, 10, 20, and 60 min and nuclei stained with Hoechst 34580 (Molecular Probes®; 1:500, 10 min). Control cells were incubated for 30 min on ice. Non-specific internalization of antibody was examined by incubating microglia with Alexa 488-labeled X63 antibody (60 min, 37°C). The average green fluorescence was measured in the perinuclear region of each cell after background subtraction. Three independent experiments were performed (*n* ≥ 77 cells per condition). Antibody internalization was quantified (ImageJ) as the increase in intracellular fluorescence by accumulated OX42 antibody in the perinuclear region. One-way analysis of variance (one-way ANOVA) and *post hoc* one-sample *t*-tests with Bonferroni correction were used to assess significance.

### TRAFFICKING OF OX42 ANTIBODY

Isolated microglial cells on fluorodishes were incubated with 2 μg/mL OX42-Alexa 488 for 30 min on ice to saturate membrane CD11b. Cells were washed to remove unbound antibody and internalized antibody chased for further 60 min at 37°C. Cell nuclei (Hoechst) and acidic vesicles (Lysotracker Red, 50 nM, Molecular Probes®;) were stained for 5 min at 37°C, extracellular fluorescence quenched with 0.2% trypan blue and cells imaged. Two experiments were performed and results assessed qualitatively.

### OX42-IMMUNOPORTER CONJUGATION

OX42 antibody in HEPES-buffered saline (HBS), pH 7.9, was modified with 10 mM *N*-Succinimidyl 3-(2-pyridyldithio)-propionate NHS ester (SPDP, ThermoFisher) at a ratio of 1.3 μL SPDP/mg OX42 for 2 h at room temperature. SPDP-modified antibody was desalted on PD-10 columns, protein positive fractions identified (DC-Protein Assay, Bio-Rad), pooled and antibody concentration estimated on a spectrophotometer (280 nm). Absorbance of the leaving group pyridine-2-thione was measured at 343 nm to calculate conjugation efficiency at the end of the immunoporter synthesis.

Branched 25 kDa PEI (Sigma) was dissolved in ultrapure water, neutralized with concentrated HCl, and desalted and buffer exchanged on PD-10 columns to HBS, pH 7.9. PEI-containing fractions were pooled and the PEI-concentration estimated with a TNBS-assay (2,4,6-trinitrobenzene sulfonic acid, Sigma) described by Snyder and Sobocinski (1975) and adapted for 96-well microplates.

Neutralized PEI was engrafted with PEG by incubating PEI with a 7.5 molar excess of 125 mM NHS-activated TMS(PEG)_12_ (ThermoFisher) for 1 h at room temperature under nitrogen atmosphere. Under these reaction conditions, 12 PEG molecules bind to 1 molecule of PEI (data not shown). PEI–PEG was then purified on a PD-10 desalting column and PEI content estimated with a TNBS-assay.

PEI–PEG was modified with SPDP by incubating PEI–PEG in HBS (pH 7.9) with 5 μL of 10 mM SPDP per milligram PEI–PEG for 1 h at room temperature. SPDP-modified PEI–PEG was desalted on a PD-10 column and an excess of 150 μL of 25 mg/mL dithiothreitol (DTT, Sigma) added to activate SPDP-modified PEI–PEG. The mixture was incubated for 1 h at room temperature under nitrogen atmosphere. After reaction, the mixture was desalted to remove cleaved pyridine 2-thione and DTT. PEI-concentration was estimated with a TNBS-assay. Activated SPDP-modified PEI–PEG were prepared immediately before conjugation to avoid extended exposure to air and oxidization of the reactive sulfhydryl-group.

SPDP-modified OX42 antibody and activated SPDP-modified PEI–PEG were incubated overnight at a molar ratio of OX42 to PEI–PEG of 1:3. Incubation was done at room temperature under nitrogen in presence of 0.1 mM ethylenediaminetetraacetic acid (EDTA, Sigma). After incubation, the reaction mixture was desalted and protein-positive (280 nm) as well as pyridine-2-thione positive fractions (343 nm) pooled separately.

Conjugated OX42-immunoporter was purified in a first step with a HiTrap SP/HP cation exchange column (GE Healthcare) using increasing molarities of salt (0.5–3 M NaCl). The eluted OX42-immunoporter conjugate was then purified in a second step on a HiLoad 16/60 Superdex 200 gel filtration column (GE Healthcare) connected to a FPLC (BioLogic DuoFlow, Bio-Rad). The final bioconjugate was concentrated with 100 kDa ultrafiltration spin columns (Millipore) to 2–3 mg/mL in HBS, pH 7.3.

The average molar ratio of conjugated OX42 antibody and PEI–PEG was calculated based on the release of pyridine-2-thione according to the manufacturer’s instructions and found to be *n* = 1.1. The yield of conjugated OX42-immunoporter was 40%.

### PLASMID DNA CLONING AND PURIFICATION

Plasmid DNA was cloned and subcultured in competent DH5α *Escherichia coli* cells (Life Technologies) using standard procedures. An enhanced EGFP-expressing plasmid (pEGFP-N1, 4.7 kb) was a gift from Dr. W. Kruger (RMIT University, Melbourne) and was purified with NucleoBond®; PC 2000 Kit (Macherey-Nagel) according to the manufacturer’s instructions. The purity of plasmid preparations was A_260/280_ ≥ 1.9 for all preparations and the integrity of sub-cloned pEGFP-N1 was confirmed by restriction enzyme analysis. A control vector pcDNA3.1/Zeo(+; 5.0 kb) which lacks the EGFP gene was a gift from Dr. H. Cuny (RMIT University, Melbourne).

### GEL RETARDATION ASSAYS

The ability of PEI polyplexes to bind DNA (400 ng/well) was tested at nitrogen/phosphate (N/P) ratios of 2–10. The N/P-ratio was calculated by taking into account the molar concentration of nitrogen residues (23.2 mmol/L) of 25 kDa branched PEI and a phosphate content of 3 nmol per 1 μg nucleic acid. PEI-conjugates and pDNA (0.1 mg/mL in HBS, pH 7.3) were mixed to form PEI-pDNA complexes and incubated at room temperature for 30 min. Gel loading buffer (10X BlueJuice^TM^, Life Technologies) was added and samples run on a 0.8% agarose gel (Promega) for 40–60 min at 100 V. Images were acquired on a fluorometer after DNA staining (SYBR®; Safe DNA, Life Technologies).

### POLYPLEX PREPARATION AND TRANSFECTION EXPERIMENTS

PEI–PEG and OX42-immunogene polyplexes were prepared at an N/P-ratio of 4. Transfectants (0.5 mg/mL in HBS) were added slowly to pDNA (0.5 mg/mL in HBS) and incubated for 15 min without vortexing. For *in vitro* transfections (20 μg DNA per well), complete cell culture medium (DMEM with 10% FBS) was then added to a total volume of 0.5 mL and the solution mixed by pipetting up and down. After removing the astrocyte-conditioned medium, mixed glia cultures were incubated with polyplexes for 16–24 h. For transfections performed in the presence of 100 μM chloroquine (Sigma), transfection medium was removed after 4 h. After removal of transfectants, a 1:1 mixture of fresh complete medium and astrocyte conditioned medium was added and cells were fixed (4% PFA, 10 min) after a total of 72 h and immunostaining performed.

Polyplexes were injected into three different brain regions of individual Sprague-Dawley rats (300–350 *g*). PEI–PEG and OX42-immunogene polyplexes were injected into the PVN (200 ng DNA; left side: PEI–PEG, right side: OX42-immunogene) or dorsal striatum (300 ng DNA). Three microgram of DNA were injected with the OX42-immunogene into the right lateral ventricle. Rats were perfused after 72 h and brain sections obtained as outlined in Section “Cellular Distribution of CD11b *In Vivo*” and IHC performed.

Transfection efficiency and specificity of PEI–PEG and OX42-immunogene *in vitro* was assessed by cell counting and was based on cell type specific markers. Values were expressed as mean ± standard error of mean (SEM) and significance assessed with either a Student’s *t*-test or one-way ANOVAs with *post hoc t*-tests with Bonferroni correction or *post hoc* one-sample *t*-tests with Bonferroni correction.

### STUDY OF OX42-IMMUNOGENE AGGREGATION BY DYNAMIC LIGHT SCATTERING

The size-distribution profile of the OX42-immunogene polyplexes was investigated by adding complete cell culture medium (DMEM with 10% FBS), OX42-immunoporter (10.3 μg) and pDNA (20 μg, to form OX42-immunogene) in this order to the measurement vial used for DLS. The effect of diluents on OX42-immunogene aggregation was determined by adding HBS, DMEM (+FBS) or DMEM (–FBS) after polyplex maturation in HBS or DMEM (+FBS).

Raw data was acquired for 30 s for each measurement (three measurements per experiment) with a Fast DLS analyser (ALV GmbH, Germany). Intensity weighted size-distribution profiles were obtained from correlation functions using ALV-Correlator Software (ALV GmbH). The hydrodynamic diameter with the highest intensity for each experiment was obtained by averaging the values from the three separate measurements. Polyplexes were prepared twice for each experimental condition.

### MEASUREMENT OF REACTIVE OXYGEN SPECIES PRODUCTION

A 96-well plate assay was developed to measure ROS production using the ROS-sensitive dye CM-H_2_DCFDA [5-(and-6)-chloromethyl-2′,7′-dichloro-dihydro-fluorescein diacetate, acetyl ester; Molecular Probes®;] which responds to intracellular oxidation with increase in fluorescence of its product DCF. Microglia were loaded with 5 μM dye in Krebs-HEPES-buffer (KHB) and incubated for 30 min (37°C). One microgram of OX42 antibody and non-viral vehicles (N/P = 4) were added to the wells and incubated at 37°C. Particulate zymosan A (Sigma, 1.5 μg/well) was used as positive control to trigger the respiratory burst and the NADPH oxidase inhibitor diphenyliodonium (DPI, Sigma) was used at 1 μM to abolish ROS production. Fluorescence was read on a plate reader (FlexStation 3) after 60 min. Baseline fluorescence values (KHB only) were subtracted and results expressed as percentage of the response to 100 ng/mL of the protein kinase C (PKC) activator phorbol-12,13-dibutyrate (PDBu, Enzo Life Sciences) which was used to normalize the responsiveness of different batches of isolated microglial cells. Results were expressed as mean ± SEM (*n* = 4–7 experiments in quadruplicate measurements) and significant ROS production was assessed with a one-way ANOVA test using *post hoc* one-sample *t*-tests with Bonferroni correction.

## RESULTS

### INTEGRIN CD11b IS EXPRESSED IN MICROGLIA BUT NOT IN ASTROCYTES

**FIGURE 1:**
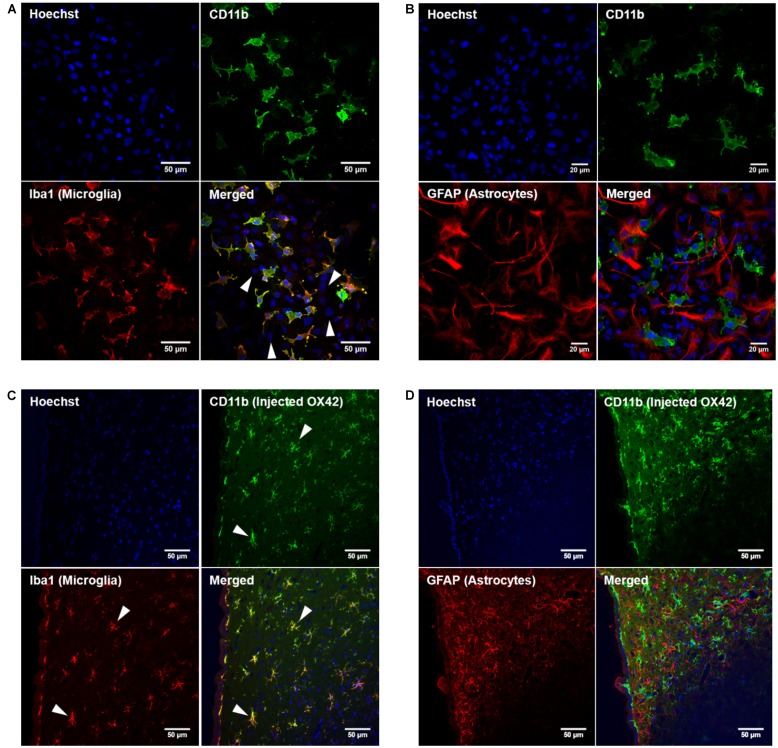
**Integrin receptor CD11b is specific for microglia *in vitro* and *in vivo*. (A)** CD11b-positive cells (green) in mixed glia culture stained for the selective microglia marker Iba1 (red). Many cells that stained for the nuclear dye Hoechst were negative for CD11b and Iba1 (white arrow heads). **(B)** GFAP-positive cells (red) did not immunostain for CD11b (green) *in vitro*. **(C)** Immunohistochemistry of rat brains injected with OX42-Alexa 488 (green) demonstrated co-labeling with microglial marker Iba1 (red, white arrow heads). **(D)** GFAP-IR (red) and CD11b-IR (green) did not co-localize showing that astrocytes do not express CD11b or take up OX42 antibody.

In order to develop microglia-specific vehicles for gene delivery with the OX42 antibody, we first investigated the cellular distribution of the target receptor CD11b. CD11b-immunreactive cells in mixed culture stained positive for Iba1 protein (**Figure 1A**). However, cells that expressed GFAP protein were not positive for CD11b (**Figure 1B**). This confirmed that CD11b expression is specific for microglia *in vitro* and that OX42 antibody binds to microglial cells only.

When Alexa 488-tagged OX42 and X63 antibodies where injected into rat brain, OX42-Alexa 488 showed strong green fluorescence and labeled many cells that appeared to be non-activated microglia with small cell bodies and long, fine processes (**Figures 1C,D**). However, X63-Alexa 488 fluorescence was only visible along the needle track and this control antibody did not bind to cells distant to the injection site (data not shown). Immunohistochemistry for microglia and astrocytes showed that the injected OX42 antibody specifically bound to microglial cells, because CD11b-positive cells also immunostained for Iba1 (**Figure 1C**). However, CD11b-IR did not co-localize with GFAP-positive astrocytes (**Figure 1D**). Thus, the *in vivo* data confirmed the specificity of CD11b for microglia, consistent with results obtained from immunofluorescence studies in cultured cells.

### OX42 IS INTERNALIZED INTO MICROGLIA VIA CD11b RECEPTOR AND TRAFFICKED TO LYSOSOMES

**FIGURE 2:**
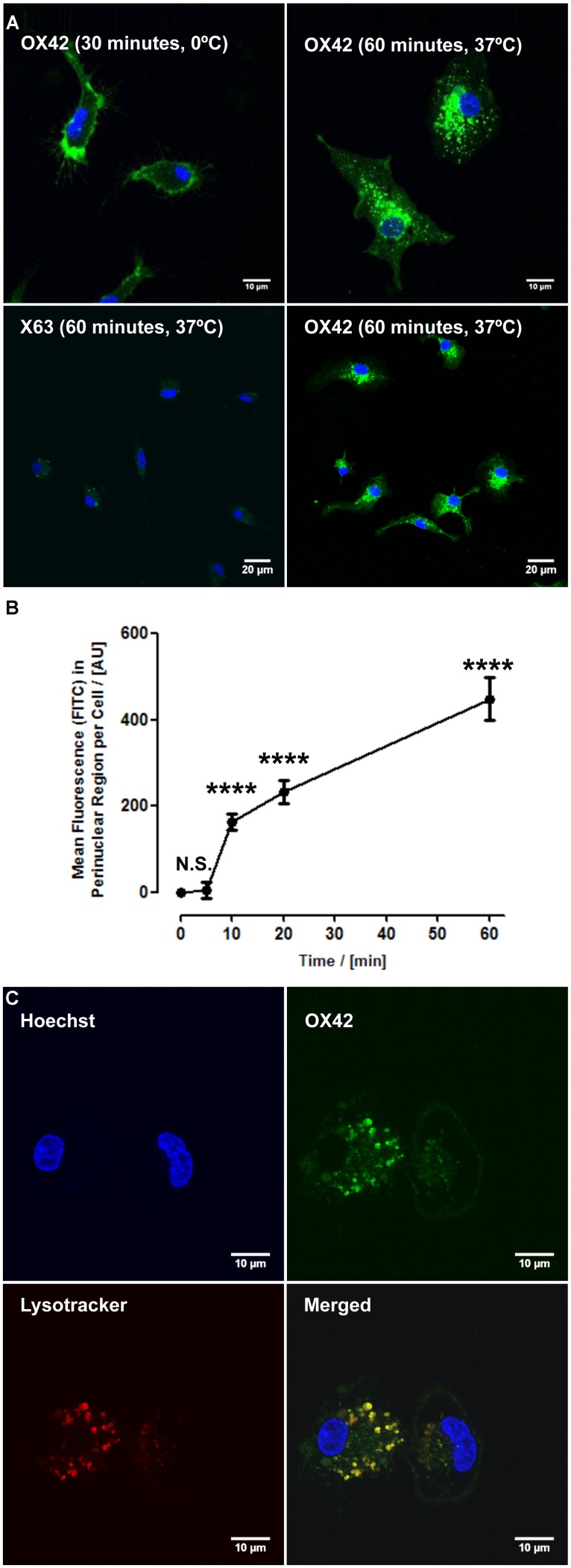
**OX42 antibody is internalized into microglia and accumulates in perinuclear acidic vesicles. (A)** Microglial cells incubated on ice with OX42-FITC (green) immunostained for CD11b in the membrane only. Microglia incubated with OX42-FITC at 37°C showed green fluorescent vesicles close to the cell nucleus (Hoechst-dye, blue) indicating the uptake of OX42 antibody into microglia. The negative control antibody X63 did not bind and internalize in microglia. **(B)** Quantification of FITC fluorescence demonstrated a rapid increase and accumulation of OX42 antibody in the perinuclear region of microglia. **(C)** The internalization of OX42 antibody was confirmed by quenching the extracellular fluorescence with trypan blue. Confocal images revealed that OX42 antibody accumulates in perinuclear acidic vesicles in microglial cells as observed by the co-localization of the acidic organelle marker Lysotracker Red. Values are plotted as Mean ± SEM. *n* ≥ 77 cells per time-point. ****P < 0.0001 vs. control; N.S., not significant; AUs, arbitrary units.

While specificity of an antibody is an important aspect for non-viral gene delivery, internalization of OX42 is required to deliver genes into the cell. Live cell experiments demonstrated that strong vesicle-like fluorescence accumulated in the perinuclear region of isolated microglial cells (**Figure 2A**). As a control, microglia were incubated with OX42-FITC on ice to halt internalization. Microglia exhibited CD11b-IR only in the cell membrane under this condition (**Figure 2A**) confirming that OX42 binds to an extracellular epitope of CD11b. Alexa 488-tagged X63 antibody did not bind nor was internalized into microglia after 60 min (**Figure 2A**) suggesting that the uptake of OX42 antibody is mediated by the CD11b receptor and does not occur via other receptors, for instance Fcγ-receptors, or other non-specific mechanisms such as pinocytosis.

Internalization of OX42 antibody was further investigated in a time-lapse experiment. After the initial 5 min incubation period, OX42-FITC fluorescence in the perinuclear region increased rapidly and significantly (10 min: 163 ± 18 AU; 20 min: 232 ± 26 AU; 60 min: 448 ± 50 AU; *P* < 0.0001; **Figure 2B**).

In order to investigate the intracellular fate of internalized OX42 antibody, internalization experiments were repeated with Alexa 488-tagged OX42 antibody due to the pH-dependence of FITC fluorescence. Microglial cells were co-labeled with Lysotracker Red, a dye that accumulates in acidic vesicles and is often used to visualize lysosomes. OX42-Alexa 488 antibody accumulated in perinuclear vesicles after 60 min (**Figure 2C**) as observed in previous internalization assays. Antibody-containing vesicles were essentially all positive for Lysotracker Red staining demonstrating that internalized OX42 antibody is trafficked to acidic organelles that most likely represent late endosomes and lysosomes. Quenching the extracellular fluorescence further confirmed that the observed perinuclear fluorescence originates from intracellular vesicles (**Figure 2C**).

In summary, these experiments demonstrated that the OX42 antibody may be a suitable ligand for non-viral gene delivery into microglia. Thus, a microglia-targeting OX42-immunoporter was developed by conjugating the OX42 antibody to PEG-engrafted PEI (PEI–PEG) via the hetero-bifunctional linker SPDP.

### THE OX42-IMMUNOPORTER BINDS PLASMID DNA

**FIGURE 3:**
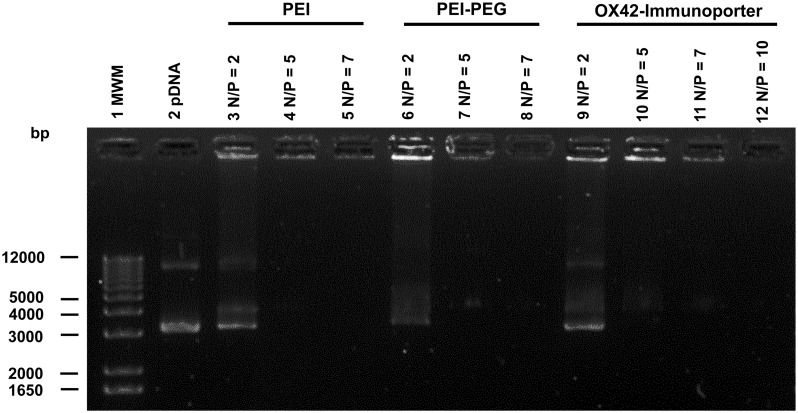
**Gel retardation assay demonstrates the ability of the OX42-immunoporter in binding and retarding plasmid DNA.** Non-linearized pDNA alone (lane 2) migrated in two main bands. PEI (lanes 3–5) and PEI–PEG (lanes 6–8) completely retarded plasmid DNA at N/P-ratios of ≥5. The OX42-immunoporter (lanes 9–12) bound and retarded DNA with increasing N/P-ratios forming an OX42-immunogene. PEI, polyethyleneimine; PEG, polyethylene glycol; IP, immunoporter; bps, base pairs; pDNA, plasmid DNA.

The ability of the OX42-immunoporter bioconjugate to bind pDNA and form the OX42-immunogene was tested in gel retardation assays. OX42-immunoporters as well as PEI and PEI–PEG were able to bind plasmid DNA with increasing Nitrogen (PEI) to Phosphate (plasmid DNA; N/P) ratios (**Figure 3**). The OX42-immunoporter vehicle completely retarded pDNA at N/P = 5.

### THE OX42-IMMUNOGENE REDUCES OFF-TARGET TRANSFECTION *IN VITRO*

**FIGURE 4:**
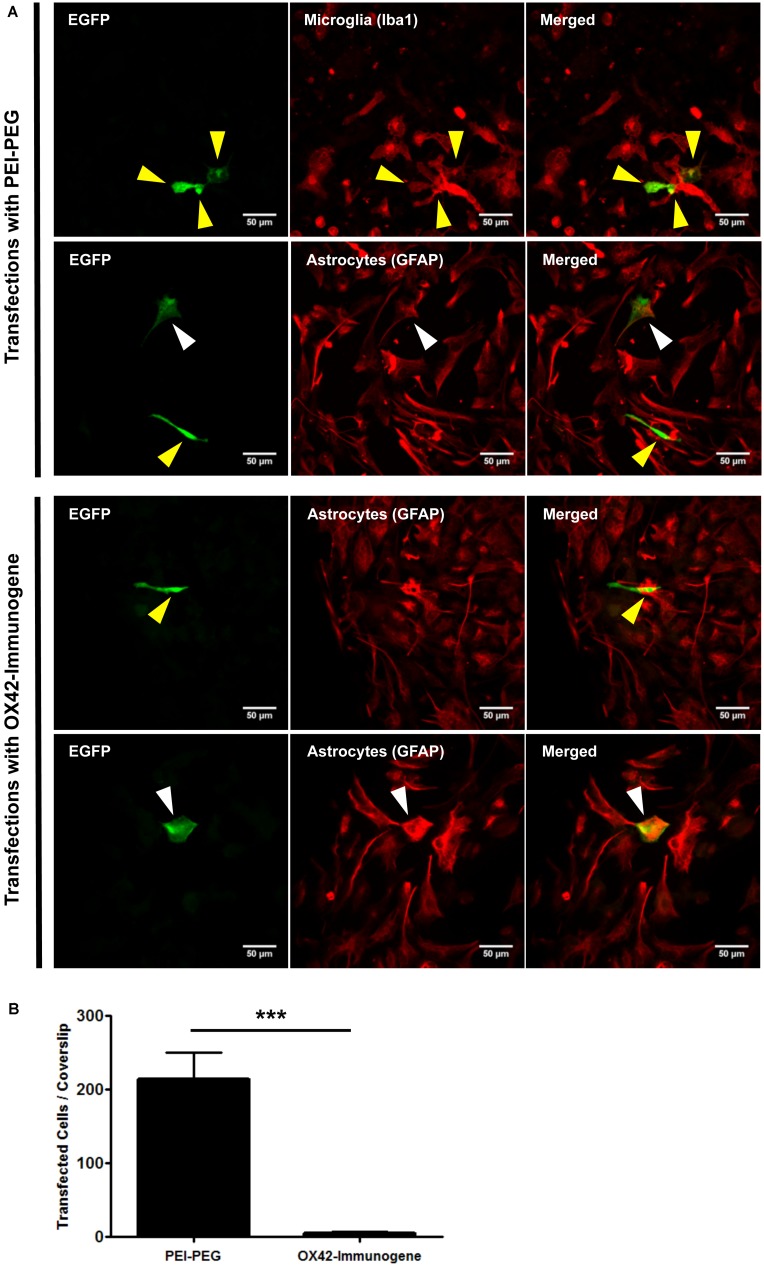
**The OX42-immunogene reduces off-target transfection of PEI–PEG *in vitro*. (A)** Representative confocal images of transfected cells in mixed culture. Non-targeting PEI–PEG (upper panel) transfected few microglial cells (yellow arrow heads) with an EGFP reporter plasmid as judged by EGFP fluorescence (green). EGFP-transfected cells were either Iba1-immunoreactive (IR, red, microglia) or lacked GFAP expression (red). PEI–PEG also transfected GFAP-IR astrocytes (white arrow heads). The targeting OX42-immunogene (lower panel) transfected few GFAP-negative cells that potentially are microglial cells (yellow arrow heads), but it also delivered the reporter gene into few GFAP-IR astrocytes (white arrow heads). **(B)** Quantification of transfected cells per coverslip. PEI–PEG (214 ± 37 cells) transfected significantly more cells than the OX42-immunogene (5 ± 2 cells). Values are plotted as Mean ± SEM. ***P < 0.005. *n* = 6 coverslips for PEI–PEG and *n* = 4 coverslips for OX42-immunogene. EGFP, green fluorescent protein; GFAP, glial fibrillary acidic protein.

Transfection efficiency and specificity of the targeting OX42-immunogene vs. non-targeting PEI–PEG were then compared in mixed glia culture. PEI–PEG transfected Iba1-IR microglia (yellow arrow heads) and GFAP-IR astrocytes (white arrow heads) as shown by EGFP-specific green fluorescence (**Figure 4A**, upper panel). The OX42-immunogene transfected very few cells *in vitro* and only some of them were potentially GFAP-negative microglia (yellow arrow heads), others were GFAP-positive astrocytes (white arrow heads, **Figure 4B**, lower panel). Quantification revealed that PEI–PEG transfected significantly more cells per coverslip than the OX42-immunogene (214 ± 37 vs. 5 ± 2, *P* < 0.005, **Figure 4B**). However, 80–90% of PEI–PEG-transfected cells were GFAP-IR astrocytes and not microglia (data not shown). Through incorporation of the OX42 antibody into the non-viral vehicle, the OX42-immunogene decreased the number of transfected cells significantly, but approximately 4 out of 5 transfected cells were still GFAP-IR astrocytes. Thus, the OX42-immunogene reduced off-target gene delivery into astrocytes, but did not cause substantial gene expression in microglia.

### INGESTION OF THE OX42-IMMUNOGENE IS ASSOCIATED WITH INCREASE OF NON-SPECIFIC FLUORESCENCE

**FIGURE 5:**
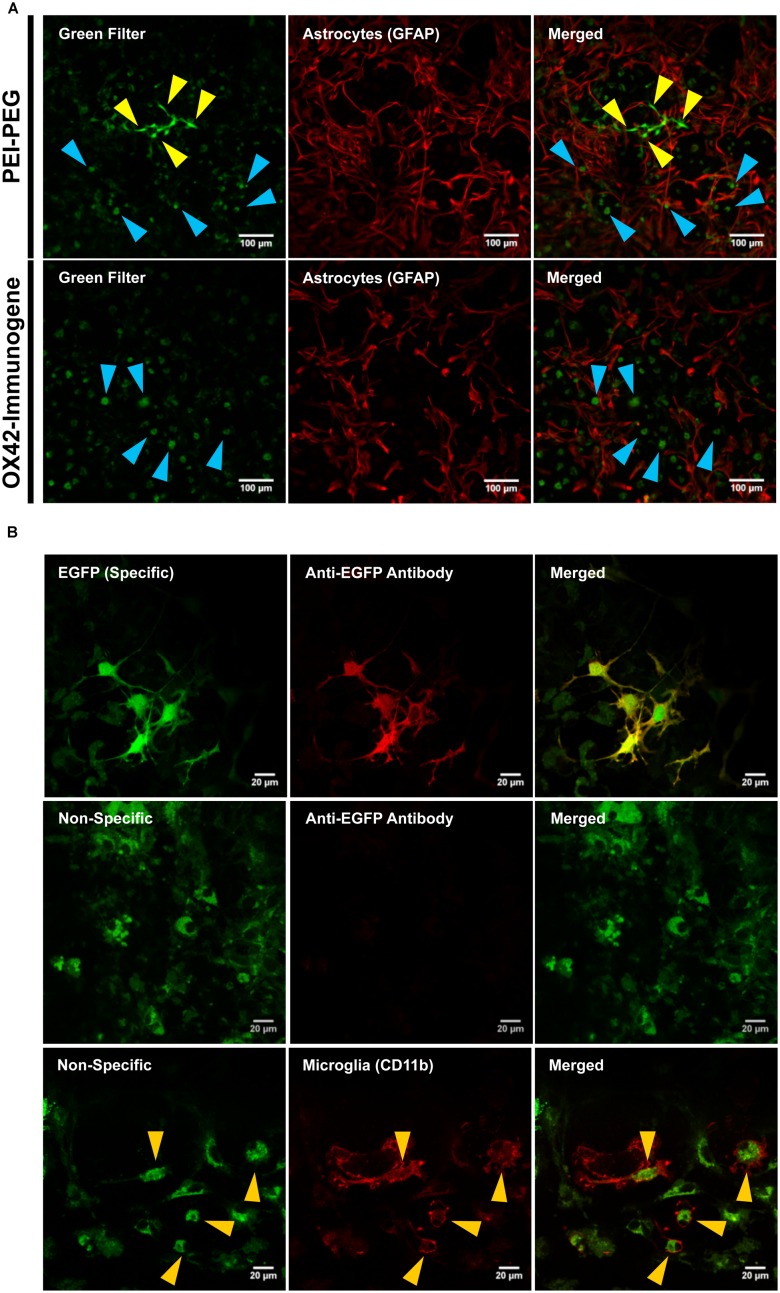
**The OX42-immunogene is taken up in microglia but does not cause gene expression *in vitro*. (A)** PEI–PEG transfected few GFAP-negative (red) cells that potentially are microglia. Green fluorescence of high (yellow arrow heads) and low intensity (blue arrow heads) was visible for mixed cultures treated with either PEI–PEG or the OX42-immunogene. The green fluorescence of low intensity almost exclusively co-localized with GFAP-negative cells (blue arrow heads). **(B)** The green fluorescence of high intensity was EGFP-specific (green) and visible in the cytoplasm and cell nucleus. Specific fluorescence caused by EGFP-expression was confirmed with an anti-EGFP antibody (red). The green fluorescence of low intensity was unrelated to EGFP-expression (non-specific) as it did not co-localize with EGFP-IR. Non-specific fluorescence (green) was mainly localized in CD11b-IR microglia (red), appeared vesicle-like and accumulated in the perinuclear region. Note: when imaging non-specific fluorescence, laser power and gain were increased compared to imaging EGFP-specific fluorescence by confocal microscopy.

The low number of cells that were transfected with the OX42-immunogene prompted an investigation of the cause of the lack of EGFP expression in microglia. Confocal images at lower magnification demonstrated that PEI–PEG caused green fluorescence of different intensities (yellow and blue arrow heads, **Figure 5A**). In OX42-immunogene treated cells, mainly green fluorescence of low intensity was observed (blue arrow heads, **Figure 5A**) and this fluorescence was almost exclusively seen in GFAP-negative microglia (**Figure 5A**).

At higher magnification and increased laser power and gain (**Figure 5B**), cells that exhibited high intensity of green fluorescence were also IR for an anti-EGFP antibody (**Figure 5B**). EGFP-specific fluorescence was distributed within the cytoplasm and cell nucleus in PEI–PEG transfected cells. In contrast, the green fluorescence of low intensity was vesicle-like, concentrated around the cell nucleus and EGFP-IR was absent (**Figure 5B**). This indicated that the non-specific fluorescence is unrelated to EGFP expression. CD11b-immunoreactive microglia were the predominant cell type that displayed non-specific fluorescence upon treatment of mixed cultures with non-viral vehicles (yellow arrow heads, **Figure 5B**).

**FIGURE 6:**
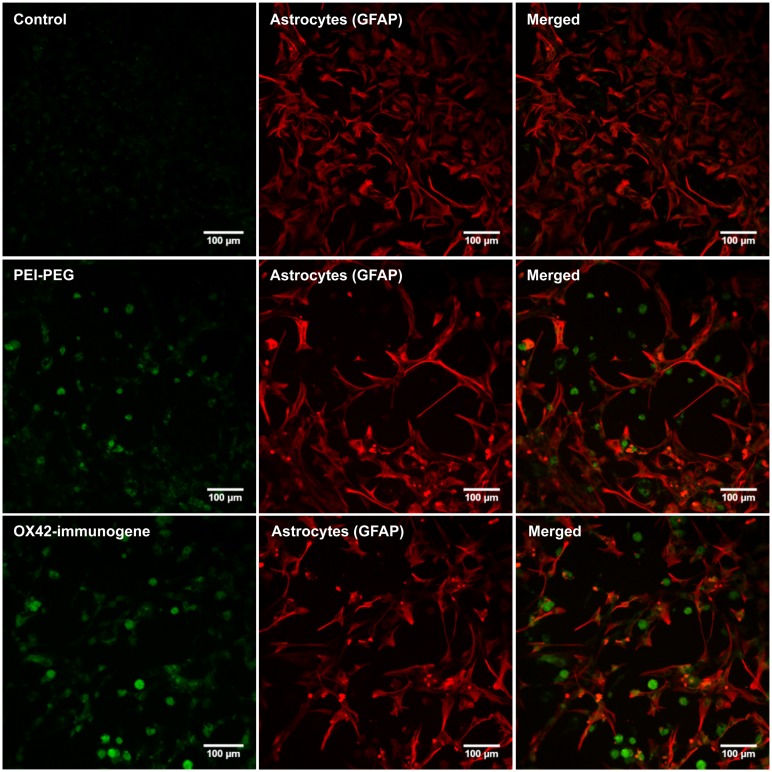
**Transfection experiments with a control vector that lacked the EGFP reporter gene caused an increase in non-specific fluorescence after treatment with PEI–PEG and the OX42-immunogene.** Untreated control mixed cultures displayed very low levels of non-specific green background fluorescence.

In order to firmly establish that the non-specific fluorescence was unrelated to EGFP-expression, mixed cultures were transfected as previously but delivering an empty control vector that lacked the EGFP reporter gene. After 3 days, PEI–PEG and OX42-immunogene treated cultures exhibited the same pattern of non-specific fluorescence (**Figure 6**) as observed in cultures treated with the transfectants carrying the EGFP-expressing plasmid (**Figures 5A,B**). This non-specific fluorescence was localized in GFAP-negative microglia, while untreated control cells had only a very low level of non-specific background fluorescence (**Figure 6**). Thus, the increase in intracellular, non-specific fluorescence was due to treatment of mixed cultures with PEI–PEG and the OX42-immunogene and it indicated that PEI–PEG and the OX42-immunogene were internalized but failed to cause gene expression in microglia

### THE OX42-IMMUNOGENE DOES NOT TRANSFECT MICROGLIA *IN VIVO*

Whether the inability of the OX42-immunogene to cause EGFP expression in microglia was an artifact of the *in vitro* test system or reflected the actual inability of the OX42-immunogene to transfect microglia was investigated *in vivo* by injecting PEI–PEG and the OX42-immunogene into different brain areas.

**FIGURE 7:**
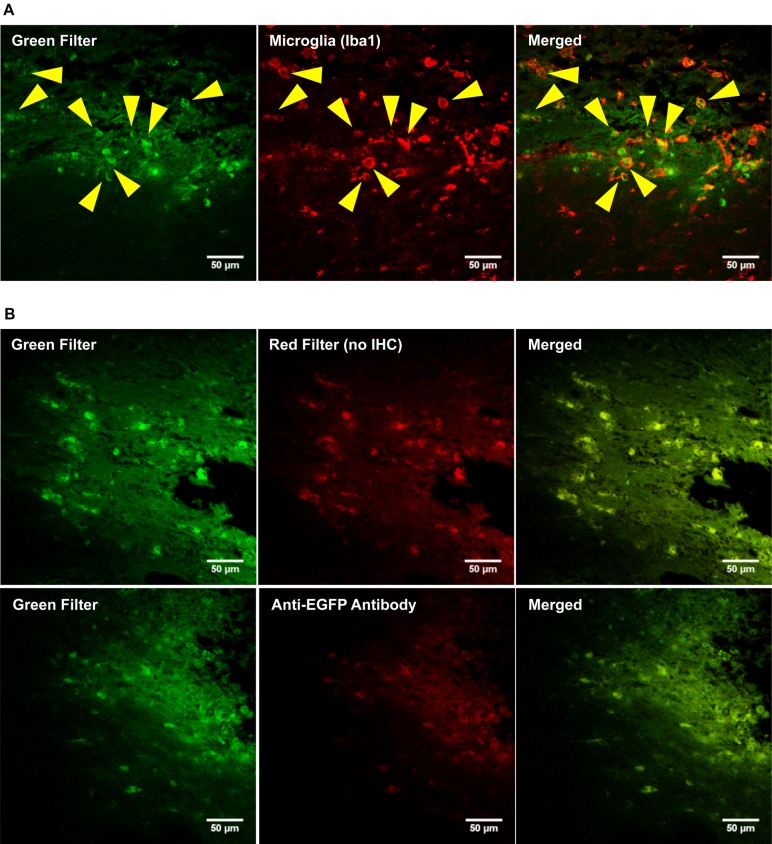
**The OX42-immunogene does not cause gene expression *in vivo*. (A)** Representative confocal images show the OX42-immunogene injected into the right lateral ventricle caused an increase in green fluorescence that co-localized in Iba1-IR microglia which exhibited an amoeboid shape (yellow arrow heads). **(B)** Confocal imaging of brain sections (dorsal striatum) revealed that the green fluorescence observed is non-specific, because non-specific fluorescence was also seen in the red filter in absence of red fluorophore-labeled cell markers. Further, an anti-EGFP antibody did not detect EGFP-expression.

PEI–PEG and the OX42-immunogene injected into the PVN (data not shown) caused an increase in green fluorescence as observed after injections of the OX42-immunogene into the right lateral ventricle (**Figure 7A**) and the dorsal striatum (**Figure 7B**). The majority of cells displaying green fluorescence were Iba1-IR microglial cells (yellow arrow heads, **Figure 7A**) that acquired a round amoeboid or phagocytic shape. Astrocytes did not exhibit green fluorescence (data not shown). The green fluorescence seen in microglia appeared to be non-specific as judged by the vesicle-like structure and absence of fluorescence in the nucleus as observed in EGFP-transfected cells *in vitro* (**Figure 5B**). After OX42-immunogene injection into the dorsal striatum, absence of EGFP-expression was demonstrated in brain sections either imaged directly without performing IHC or immunostaining with a mouse anti-EGFP antibody (**Figure 7B**). In absence of cell type specific markers, the fluorescence observed was non-specific and also seen in the red filter. Further, EGFP-IR was not observed either (**Figure 7B**) demonstrating that EGFP was not expressed *in vivo*. Further, the absence of an increased red fluorescence due to the secondary anti-mouse antibody also suggested that the OX42-immunogene was degraded, because the secondary antibody did not cross-react with the OX42 antibody component of the immunogene. Thus, the absence of EGFP-expression in microglia *in vivo* was consistent with the lack of EGFP-expression *in vitro*.

### OX42-IMMUNOGENE AGGREGATES TRIGGER THE RESPIRATORY BURST IN MICROGLIA

**FIGURE 8:**
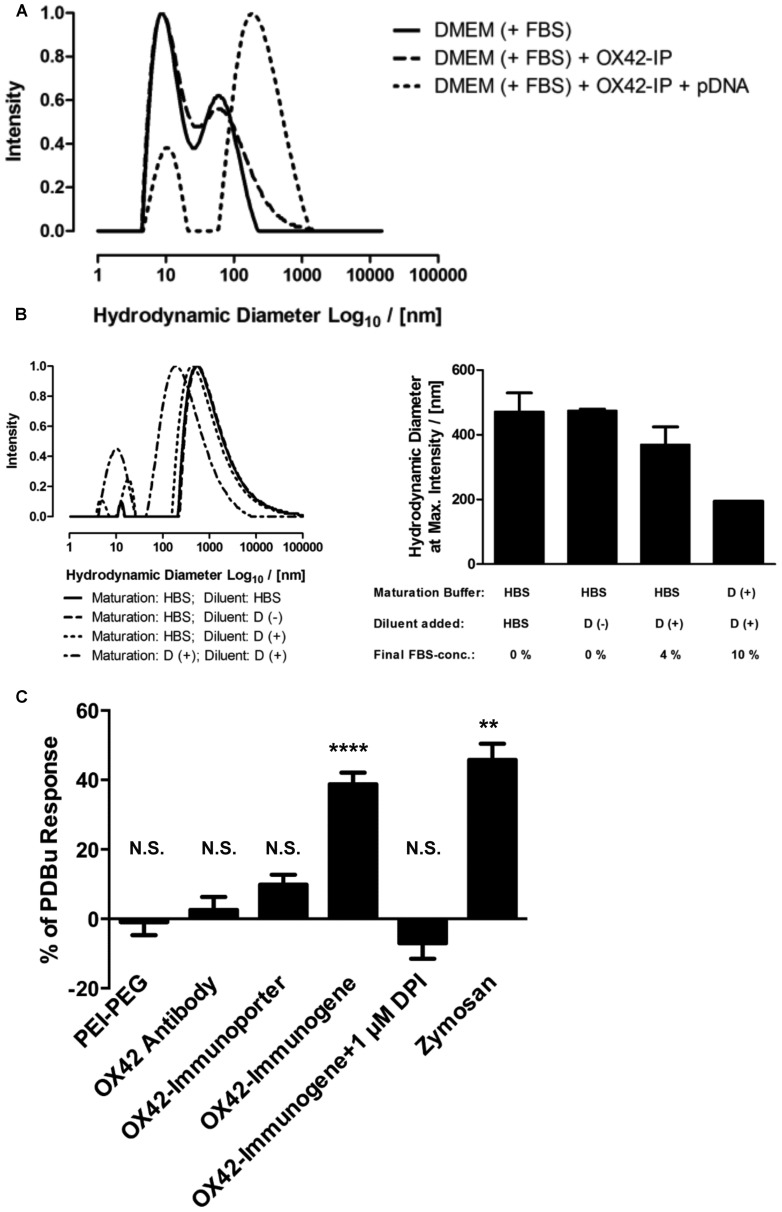
**The OX42-immunogene forms large aggregates and triggers an immune response in microglia. (A)** Comparison of the size-distribution profiles between complete cell culture medium (DMEM + 10% FBS) and the OX42-immunoporter obtained by DLS shows that the non-viral vehicle did not form aggregates. After adding pDNA to form the OX42-immunogene (N/P = 4), aggregates formed over a large range (≈50–1300 nm). **(B)** Adding FBS to polyplexes after maturation in HBS and maturation of polyplexes in FBS-containing medium caused a shift in size-distribution to lower aggregate sizes, but it had no effect on the width of size-distributions. Values are plotted as Mean ± SEM (two experiments). FBS, fetal bovine serum; OX42-IP, OX42-immunoporter; HBS, HEPES-buffered saline; D (+/–), DMEM with/without 10% FBS. **(C)** ROS production in microglial cells was measured by quantifying ROS-indicator fluorescence after 60 min and reported as percentage (%) of the internal standard PDBu. The aggregated OX42-immunogene triggered the respiratory burst (38.8 ± 3.3%) which was inhibited by the respiratory burst inhibitor diphenyliodonium (–7.02 ± 4.49%). The positive control zymosan caused the release of ROS at similar levels (45.8 ± 4.7%) to the OX42-immunogene. However, PEI–PEG (–0.97 ± 3.74%), OX42 antibody (2.57 ± 1.68%) and the OX42-immunoporter (9.83 ± 2.84%) did not trigger significant ROS production. Values are plotted as Mean ± SEM. **P < 0.01 vs. control; ****P < 0.0001 vs. control; N.S., not significant. DPI, diphenyliodonium; PDBu, phorbol 12,13-dibutyrate.

The uptake of the non-viral vehicles into microglia, the apparent intracellular degradation concomitant with increase in non-specific fluorescence and the absence of widespread gene expression in microglia prompted an investigation into the reasons of this phenomenon. Initial experiments aimed at determining the size-distribution profiles for the OX42-immunogene formed in complete cell culture medium (DMEM with 10% FBS, **Figure 8A**). Complete cell culture medium contained small aggregates of <100 nm in diameter (**Figure 8A**). There was no marked difference in the size-distribution profile when the OX42-immunoporter was added suggesting that the vehicle had not substantially aggregated. However, when pDNA was added to form the OX42-immunogene complex (N/P = 4), large OX42-immunogene aggregates formed over a wide diameter range (≈50–1300 nm, **Figure 8A**). Based on the aggregate sizes observed, this suggested that the OX42-immunogene could potentially be internalized via receptor-mediated endocytosis, but larger aggregates could trigger phagocytosis in microglia.

Further experiments were designed to understand how the aggregation behavior of the OX42-immunogene depended on sample preparation. The mode of OX42-immunogene aggregate size was found to be strongly dependent on FBS, either added as diluent after maturation of complexes in HBS or already present in the maturation buffer (**Figure 8B**). Although FBS caused a shift in the size-distribution profile to smaller aggregates, it did not markedly narrow the size-distribution (**Figure 8B**). DLS results further suggested that the absence of FBS for *in vivo* transfections would cause the formation and injection of larger aggregates as compared to *in vitro* transfections where the OX42-immunogene was formed in HBS, but FBS-containing cell culture medium was added to a final concentration of 9% FBS in the transfection medium.

The consequences of microglial phagocytosis of aggregated polyplexes were then investigated by measuring the production of ROS. There was a significant increase in the observed fluorescence of the ROS indicator in microglia treated with the OX42-immunogene (38.8 ± 3.3%, *P* < 0.0001; **Figure 8C**). DPI (1 μM), an inhibitor of the respiratory burst, abolished ROS production in microglia elicited by the aggregated OX42-immunogene (–7.02 ± 4.49%). Non-targeting PEI–PEG did not stimulate ROS production (–0.97 ± 3.74%) suggesting that the respiratory burst caused by the immunogene was mediated by specific receptors on the microglial membrane.

Further, aggregation of the OX42-immunogene as seen by DLS (**Figures 8A,B**) was crucial to trigger the respiratory burst, because OX42 antibody alone (2.57 ± 1.68%) and the non-aggregated OX42-immunoporter stimulated only low levels of ROS production (9.83 ± 2.84%). Particulate zymosan as a positive control generated significant levels of ROS (45.8 ± 4.7%, *P* < 0.01) similar to the aggregated OX42-immunogene. Therefore, the aggregated OX42-immunogene triggered a strong inflammatory response in microglia as demonstrated by a phagocytosis-induced respiratory burst.

### CHLOROQUINE FACILITATES ENDOSOMAL ESCAPE IN MICROGLIA

Dynamic light scattering data and the ROS assay showed that the OX42-immunogene formed large complexes with the ability to trigger phagocytosis and an unwanted immune response that potentially is linked to intracellular destruction of the immunogene. However, this data did not explain why non-targeting PEI–PEG in absence of the respiratory burst did not cause gene expression in microglia, although PEI–PEG was taken up by microglial cells (). Thus, we examined limited endosomal escape as an additional barrier to non-viral gene delivery into microglia.

**FIGURE 9:**
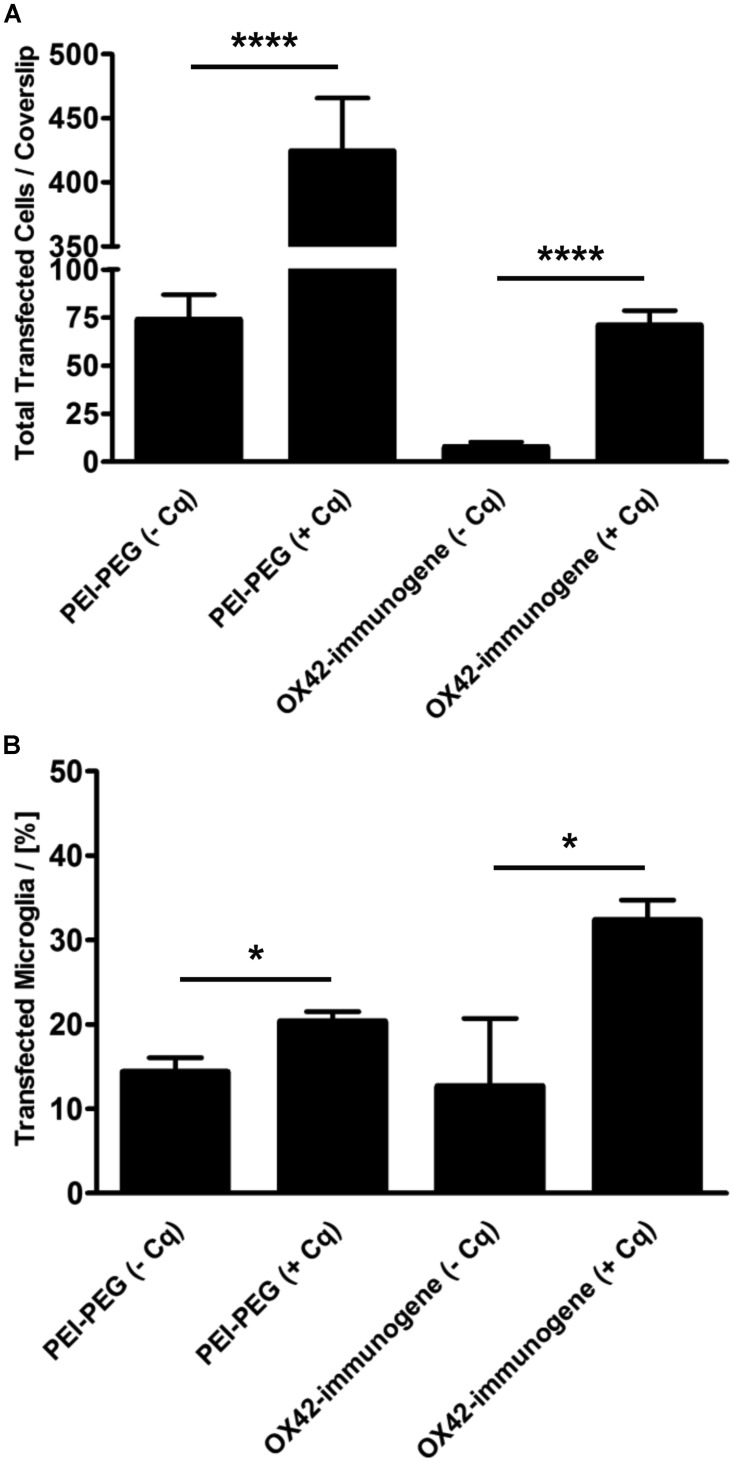
**The ability of PEI to facilitate intracellular escape in microglia is limited. (A)** PEI–PEG (424 ± 42 vs. 74 ± 13) and the aggregated OX42-immunogene (71 ± 8 vs. 8 ± 3) transfected significantly more cells in the presence of chloroquine. **(B)** Chloroquine also increased specificity for microglia when transfected with PEI–PEG (20.3 ± 1.2% vs. 14.3 ± 1.7%) and the OX42-immunogene (32.3 ± 2.4% vs. 12.7 ± 8.0%). Values are plotted as Mean ± SEM. *n* = 6 coverslips (three independent experiments). *P < 0.05, ****P < 0.0001. Cq, chloroquine.

Mixed glia cultures were transfected with PEI–PEG and the OX42-immunogene in the presence of the endosomolytic agent chloroquine. Chloroquine treatment significantly increased the total number of cells transfected with PEI–PEG (424 ± 42 vs. 74 ± 13, *P* < 0.0001) and the OX42-immunogene (71 ± 8 vs. 8 ± 3, *P* < 0.0001) at N/P = 4 (**Figure 9A**). Interestingly, chloroquine caused a significant increase in the percentage of transfected cells for both transfectants that were microglia (PEI–PEG: 20.3 ± 1.2% vs. 14.3 ± 1.7%, *P* < 0.05; OX42-immunogene: 32.3 ± 2.4% vs. 12.7 ± 8.0%, *P* < 0.05; **Figure 9B**) demonstrating that endosomal escape is limiting for non-viral gene transfer into microglia. However, despite this increase in presence of chloroquine the majority of the cells transfected with the OX42-immunogene were still not microglia. This showed that the OX42-immunogene is not entirely specific for microglia and the data strongly suggested the involvement of another route in internalization of the OX42-immunogene other than CD11b, for instance a receptor which is shared by microglia and other glial cells.

## DISCUSSION

This study took a systematic approach at examining the use of antibody-based non-viral vehicles for microglia-specific gene transfer. Double-labeling experiments in mixed cultures were performed with OX42 antibody and markers for the two most abundant cell types in mixed glia culture – GFAP for astrocytes and Iba1 for microglia. The experiments clearly demonstrated the specificity of CD11b for microglia. This is consistent with previous reports (Akiyama and McGeer, 1990) and accounts for the use of OX42 antibody as a selective microglial marker for immunocytochemistry and IHC. *In vivo* data confirmed the specificity of OX42 antibody for microglia and the absence of X63-binding strongly suggests attachment of OX42 to microglia via the CD11b receptor. Because microglia and other cells within the CNS express Fcγ-receptors (Ulvestad et al., 1994; Vedeler et al., 1994; Okun et al., 2010) that can bind antibodies and are also capable of ingesting extracellular material by pinocytosis (Booth and Thomas, 1991; Ward et al., 1991), the absence of these alternative internalization mechanisms is essential for specific gene transfer into microglia via CD11b.

The usefulness of the OX42 antibody was further emphasized by its property to be rapidly internalized into microglia. The negative control antibody X63 did not bind and was not taken up by microglia demonstrating that internalization of OX42 must occur via the extracellular domain of the CD11b receptor. Direct immunofluorescence studies and co-labeling with Lysotracker Red, a marker for acidic late endosomes and lysosomes, further showed that the internalized antibody was trafficked to acidic organelles in the perinuclear cytoplasm. The known perinuclear localization of lysosomes and late endosomes (Huotari and Helenius, 2011) agreed with the localization of OX42-fluorescence seen here confirming lysosomal trafficking of OX42 antibody in microglia. Lysosomal trafficking is desirable for non-viral vehicles that utilize PEI as DNA condensation agent, because the gradual acidification of endosomes facilitates the endosomal escape of the gene vehicle (Varkouhi et al., 2011). The proximity of lysosomes to the cell nucleus also improves transfection efficiency by reducing the spatial distance from endosome exit to nucleus entry (Forrest and Pack, 2002).

Since we demonstrated the usefulness of the OX42 antibody as microglia-specific ligand, a bioconjugate was developed based on the OX42 antibody and branched PEI of 25 kDa engrafted with PEG. The resulting OX42-immunoporter showed complete DNA-binding at a N/P-ratio of 5 as demonstrated in gel retardation assays. Subsequent transfection experiments were carried out at N/P = 4 to avoid an excess of PEI that can cause competing non-specific uptake mechanisms via positively charged PEI (Kircheis et al., 2001; Payne et al., 2007). Further, initial transfection experiments with PEI–PEG at N/P = 10 reduced the number of transfected cells and appeared to have toxic effects in mixed glia culture as judged by the up-regulation of GFAP expression and morphology of astrocytes (data not shown).

The conjugation of the OX42 antibody to PEI–PEG caused significant reduction of off-target transfection of PEI–PEG *in vitro*. However, the OX42-immunogene transfected only few cells with no apparent increase in specificity for microglia. Expression of EGFP was mostly absent in microglia regardless of whether PEI–PEG or the OX42-immunogene was used for transfection *in vitro* and *in vivo*. This was demonstrated by the absence of EGFP-IR with an anti-EGFP antibody and by the observed pattern for green fluorescence of high intensity (EGFP, cytoplasmic and nuclear) and low intensity (non-specific, vesicle-like and perinuclear). Treating mixed glia cultures with PEI–PEG or the OX42-immunogene carrying a control vector that lacked the EGFP reporter gene then confirmed that the polyplexes were taken up by microglia and that the intracellular non-specific fluorescence is unrelated to EGFP expression. Thus, the non-specific fluorescence was thought to be related to an intracellular event downstream of internalization.

Previous work showed that cultured microglial cells exhibit non-specific fluorescence when treated with extracellular material destined for intracellular degradation (Stolzing et al., 2002). Importantly, microglia were shown to degrade only mildly oxidized protein efficiently while strongly oxidized proteins were accumulated intracellular and exhibited broad-spectrum auto-fluorescence (Stolzing et al., 2002). Incompletely degraded material that causes non-specific fluorescence has been termed “lipofuscin” (Sitte et al., 2000; Stolzing et al., 2002; Lei et al., 2012). The increase in non-specific, lipofuscin-like fluorescence in microglia therefore most likely originates from internalization and accumulation of incompletely degraded PEI–PEG and OX42-immunogene. The perinuclear localization of this vesicle-like, non-specific fluorescence suggests that microglia degrade the polyplexes in lysosomes. The observed amoeboid, phagocytic shape of microglia *in vivo* further indicates that microglia internalize the polyplexes by phagocytosis rather than receptor-mediated endocytosis.

Dynamic light scattering showed that the OX42-immunogene formed a heterogeneous population of aggregated polyplexes from less than 100 nm to more than 1 μm in diameter. This demonstrated that phagocytosis is one of the mechanisms by which the OX42-immunogene can be internalized. The stimulation of a respiratory burst demonstrated that the aggregated OX42-immunogene but not PEI–PEG and the OX42 antibody alone triggers a strong inflammatory response in microglia. Clearly, stimulation of an immune response is unwanted not only because this most likely trigger functions in microglia associated with their role as scavengers and cause destruction of the OX42-immunogene, but also because of potential exacerbation of neurodegeneration.

The data on ROS generation further gave evidence that the phagocytosis-induced respiratory burst not only requires aggregated large particles, but also receptors on the cell surface. Since the OX42-immunogene was developed to target CD11b on microglia and to undergo receptor-mediated endocytosis, aggregation of the OX42-immunogene most likely caused the stimulation of other receptors such as FcγR which require receptor cross-linking by antibody-coated targets (reviewed in Nimmerjahn and Ravetch, 2008). The OX42-immunogene may have mimicked antibody-coated immune complexes cleared by the phagocytic system of microglial cells. This could have occurred via phagocytosis receptors including CD11b and Fcγ-receptors as previously reported for macrophages (Thornton et al., 1996; Xia et al., 1999; Huang et al., 2011) that perform functions closely related to microglial cells.

Previous work on peripheral immune cells related to microglia revealed that CD11b and FcγRs interact to facilitate the rate of phagocytosis (Mantovani et al., 1972; Mantovani, 1975; Jongstra-Bilen et al., 2003; Huang et al., 2011). This observation becomes even more important when the binding site of OX42 on CD11b is considered. The OX42 antibody is suggested to bind to, or close to, the complement-binding site of CD11b (Robinson et al., 1986; Klegeris and McGeer, 1994; Sohn et al., 2003) which is important for phagocytosis of complement-coated apoptotic cells in absence of cytotoxic immune effector functions (Amarilyo et al., 2010; Hughes et al., 2010; Ricklin et al., 2010). The initiation of a respiratory burst therefore strongly argues for the involvement of FcγRs in uptake of the OX42-immunogene. Thus, the inability of the OX42-immunogene to cause substantial gene expression in microglia may be due to targeting receptors other than CD11b and directing the gene vehicle to a less efficient gene delivery pathway for microglial cells such as FcγR-mediated phagocytosis.

While the respiratory burst elicited by the aggregated OX42-immunogene limits its use for microglial gene transfer, non-targeting PEI–PEG at N/P = 4 did not cause substantial gene expression in microglia either albeit the absence of ROS production. Interestingly, PEI–PEG was more successful in gene transfer *in vitro*, although most of the transfected cells were astrocytes. PEI–PEG did not transfect any cells *in vivo* and was predominantly taken up by microglial cells with subsequent intracellular degradation. The unsuccessful transfection of microglia with PEI–PEG *in vivo* is consistent with a previous report that used N/P-ratios as high as 15 (Kwon et al., 2010). However, the difficulty of transfecting brain cells with non-viral vehicles *in vivo* may not be limited to PEI–PEG, because a novel cationic polymer that showed high transfection efficiency in culture was able to increase gene transfer *in vivo* only slightly as compared to naked DNA (Newland et al., 2014). The phagocytic/amoeboid morphology and expression of Iba1 on the cell membrane (Ohsawa et al., 2000) further suggested that PEI–PEG also stimulated microglial activation, although through apparent different mechanisms compared to the OX42-immunogene.

Data obtained by DLS hints at the sample preparation as decisive factor for particle size and the internalization mechanism triggered. PEI-polyplexes were prepared in physiological salt solution for *in vivo* injections. However, for *in vitro* transfections these complexes were further diluted in complete cell culture medium containing FBS. In accordance with studies performed in other cell types (Ogris et al., 1998; Petersen et al., 2002; Neu et al., 2005), DLS data showed that sample preparation in general and the presence of FBS in particular shifts polyplex aggregates to smaller sizes that could favor endocytosis, at least for astrocytes. Therefore, larger aggregates may have been injected *in vivo* that shifted the internalization of PEI–PEG aggregates to professional phagocytes that take up large aggregates and destroy them such as microglia. While phagocytosis is a receptor-mediated internalization mechanism, non-specific internalization mechanisms such as macropinocytosis (Mayor and Pagano, 2007; Hufnagel et al., 2009; Xiang et al., 2012) may have occurred which is usually confined to immune cells such as macrophages and microglia (Kerr and Teasdale, 2009; Mandrekar et al., 2009; Mercer and Helenius, 2009).

The inability of PEI–PEG in this study to transfect a large number of microglia as compared to astrocytes point to different internalization mechanisms between these glia cells and the ineffectiveness of branched PEI (bPEI, 25 kDa) in promoting endosomal escape and gene transfer in microglia. This notion is further supported by the observation that in presence of chloroquine, a drug used previously to promote endosomal escape and enhance transfection efficiency of non-viral vehicles in other cell types (Erbacher et al., 1996; Ogris et al., 1998; Blessing et al., 2001), PEI–PEG and the OX42-immunogene transfected significantly more cells including microglia *in vitro*. Indeed, the success of gene transfer with PEI depends on polyplex type (linear or branched PEI, molecular weight) and the cell type transfected (Kircheis et al., 1997; Ogris et al., 1998; von Gersdorff et al., 2006; Intra and Salem, 2008) and may need to be optimized for microglial gene transfer. However, more than 60% of transfected cells in presence of chloroquine were not microglia even when cells were transfected with the OX42-immunogene. This supports the assumption that the aggregated OX42-immunogene not only targets the CD11b receptor but also FcγRs, because astrocytes and other brain cells express at least one FcγR subtype *in vitro* and *in vivo* (Nitta et al., 1992; Li et al., 2008; Okun et al., 2010).

## CONCLUSION

This study highlighted for the first time the CD11b receptor as a potential target for non-viral gene transfer into microglia with antibodies. Data presented here show that the anti-CD11b antibody OX42 is specific for microglia, is rapidly internalized, trafficked to lysosomes and does not elicit a strong immune response. This study also demonstrates that the synthesis of a microglia-targeting non-viral vehicle can be accomplished. However, the absence of substantial reporter gene expression in microglia *in vitro* and *in vivo* demonstrates that microglia is difficult to transfect with non-viral vehicles based on branched PEI of 25 kDa. While PEI–PEG and the OX42-immunogene are both internalized into microglia, they both are subject to intracellular degradation.

Two barriers to receptor-mediated gene transfer into microglia were characterized that will help to further develop second generation immunogenes targeting microglial cells. Bypassing FcγR-mediated phagocytosis and the respiratory burst will require the use of OX42-F(ab’)_2_ antibody fragments that lack FcγR-binding sites. These antibody fragments should also increase specificity toward CD11b and microglia by avoiding cross-reaction with other cells that express FcγRs. The inability of PEI–PEG to cause substantial gene expression in microglia demonstrates that endosomal escape is another barrier for non-viral gene transfer into microglia and that alternative PEI-polymers may be required that give higher levels of gene expression in microglia. This study suggests that phagocytosis is not an efficient pathway for microglial transfection. However, it remains to be established whether phagocytosis via CD11b in absence of an immune response is able to deliver genes into microglia.

## Conflict of Interest Statement

Robert A. Rush owns shares in Biosensis Pty Ltd., Adelaide, which sells the OX42 and X63 antibodies.

## References

[B1] Aguzzi A. Barres B. A. Bennett M. L. (2013). Microglia: scapegoat, saboteur, or something else? *Science* 339 156–161 10.1126/science.1227901 23307732 PMC4431634

[B2] Akinc A. Thomas M. Klibanov A. M. Langer R. (2005). Exploring polyethylenimine-mediated DNA transfection and the proton sponge hypothesis. *J. Gene Med.* 7 657–663 10.1002/jgm.696 15543529

[B3] Akiyama H. McGeer P. L. (1990). Brain microglia constitutively express β-2 integrins. *J. Neuroimmunol.* 30 81–93 10.1016/0165-5728(90)90055-R 1977769

[B4] Amarilyo G. Verbovetski I. Atallah M. Grau A. Wiser G. Gil O. (2010). iC3b-opsonized apoptotic cells mediate a distinct anti-inflammatory response, and transcriptional NF-κB-dependent blockade. *Eur. J. Immunol.* 40 699–709 10.1002/eji.200838951 20039295

[B5] Aouadi M. Tesz G. J. Nicoloro S. M. Wang M. Chouinard M. Soto E. (2009). Orally delivered siRNA targeting macrophage Map4k4 suppresses systemic inflammation. *Nature* 458 1180–1184 10.1038/nature07774 19407801 PMC2879154

[B6] Balcaitis S. Weinstein J. R. Li S. Chamberlain J. S. Moeller T. (2005). Lentiviral transduction of microglial cells. *Glia* 50 48–55 10.1002/glia.20146 15625717

[B7] Barati S. Hurtado P. R. Zhang S. H. Tinsley R. Ferguson I. A. Rush R. A. (2006). GDNF gene delivery via the p75^NTR^ receptor rescues injured motor neurons. *Exp. Neurol.* 202 179–188 10.1016/j.expneurol.2006.05.027 16842780

[B8] Berhanu D. A. Rush R. A. (2008). Targeted silencing of TrkA expression in rat forebrain neurons via the p75 receptor. *Neuroscience* 153 1115–1125 10.1016/j.neuroscience.2008.03.025 18440710

[B9] Berton G. Lowell C. A. (1999). Integrin signalling in neutrophils, and macrophages. *Cell. Signal.* 11 621–635 10.1016/S0898-6568(99)00003-0 10530871

[B10] Blessing T. Kursa M. Holzhauser R. Kircheis R. Wagner E. (2001). Different strategies for formation of PEGylated EGF-conjugated PEI/DNA complexes for targeted gene delivery. *Bioconjug. Chem.* 12 529–537 10.1021/bc0001488 11459457

[B11] Block M. L. Zecca L. Hong J. S. (2007). Microglia-mediated neurotoxicity: uncovering the molecular mechanisms. *Nat. Rev. Neurosci.* 8 57–69 10.1038/nrn2038 17180163

[B12] Bokhoven M. Stephen S. L. Knight S. Gevers E. F. Robinson I. C. Takeuchi Y. (2009). Insertional gene activation by lentiviral, and gammaretroviral vectors. *J. Virol.* 83 283–294 10.1128/JVI.01865-08 18945765 PMC2612344

[B13] Booth P. L. Thomas W. E. (1991). Evidence for motility, and pinocytosis in ramified microglia in tissue culture. *Brain Res.* 548 163–171 10.1016/0006-8993(91)91118-K 1868330

[B14] Burke B. Sumner S. Maitland N. Lewis C. E. (2002). Macrophages in gene therapy: cellular delivery vehicles, and in vivo targets. *J. Leukoc. Biol.* 72 417–428 12223508

[B15] Cucchiarini M. Ren X. L. Perides G. Terwilliger E. F. (2003). Selective gene expression in brain microglia mediated via adeno-associated virus type 2, and type 5 vectors. *Gene Ther.* 10 657–667 10.1038/sj.gt.3301925 12692594

[B16] Davalos D. Kyu Ryu J. Merlini M. Baeten K. M. Le Moan N. Petersen M. A. (2012). Fibrinogen-induced perivascular microglial clustering is required for the development of axonal damage in neuroinflammation. *Nat. Commun.* 3:1227 10.1038/ncomms2230 PMC3514498 23187627

[B17] Dominguez E. Mauborgne A. Mallet J. Desclaux M. Pohl M. (2010). SOCS3-mediated blockade of JAK/STAT3 signaling pathway reveals its major contribution to spinal cord neuroinflammation, and mechanical allodynia after peripheral nerve injury. *J. Neurosci.* 30 5754–5766 10.1523/JNEUROSCI.5007-09.2010 20410127 PMC6632340

[B18] Duan Y. Yang C. Zhang Z. Liu J. Zheng J. Kong D. (2010). Poly(ethylene glycol)-grafted polyethylenimine modified with G250 monoclonal antibody for tumor gene therapy. *Hum. Gene Ther.* 21 191–198 10.1089/hum.2009.010 19788387

[B19] Erbacher P. Bousser M. T. Raimond J. Monsigny M. Midoux P. Roche A. C. (1996). Gene transfer by DNA/glycosylated polylysine complexes into human blood monocyte-derived macrophages. *Hum. Gene Ther.* 7 721–729 10.1089/hum.1996.7.6-721 8919594

[B20] Ferkol T. Mularo F. Hilliard J. Lodish S. Perales J. C. Ziady A. (1998). Transfer of the human alpha_1_-antitrypsin gene into pulmonary macrophages *in vivo*. *Am. J. Respir. Cell Mol. Biol.* 18 591–601 10.1165/ajrcmb.18.5.2874 9569229

[B21] Ferkol T. Perales J. C. Mularo F. Hanson R. W. (1996). Receptor-mediated gene transfer into macrophages. *Proc. Natl. Acad. Sci. U.S.A.* 93 101–105 10.1073/pnas.93.1.101 8552583 PMC40186

[B22] Forrest M. L. Pack D. W. (2002). On the kinetics of polyplex endocytic trafficking: implications for gene delivery vector design. *Mol. Ther.* 6 57–66 10.1006/mthe.2002.0631 12095304

[B23] Germershaus O. Merdan T. Bakowsky U. Behe M. Kissel T. (2006). Trastuzumab-polyethylenimine-polyethylene glycol conjugates for targeting Her2-expressing tumors. *Bioconjug. Chem.* 17 1190–1199 10.1021/bc0601119 16984128

[B24] Griffiths M. R. Gasque P. Neal J. W. (2009). The multiple roles of the innate immune system in the regulation of apoptosis, and inflammation in the brain. *J. Neuropathol. Exp. Neurol.* 68 217–226 10.1097/NEN.0b013e3181996688 19225414

[B25] Hajishengallis G. Lambris J. D. (2011). Microbial manipulation of receptor crosstalk in innate immunity. *Nat. Rev. Immunol.* 11 187–200 10.1038/nri2918 21350579 PMC3077082

[B26] Hu X. Zhang D. Pang H. Caudle W. M. Li Y. Gao H. (2008). Macrophage antigen complex-1 mediates reactive microgliosis, and progressive dopaminergic neurodegeneration in the MPTP model of Parkinson’s disease. *J. Immunol.* 181 7194–7204 10.4049/jimmunol.181.10.7194 18981141 PMC2759089

[B27] Huang Z. Y. Hunter S. Chien P. Kim M. K. Han-Kim T. H. Indik Z. K. (2011). Interaction of two phagocytic host defense systems: Fcγ receptors, and complement receptor 3. *J. Biol. Chem.* 286 160–168 10.1074/jbc.M110.163030 21044955 PMC3012970

[B28] Hufnagel H. Hakim P. Lima A. Hollfelder F. (2009). Fluid phase endocytosis contributes to transfection of DNA by PEI-25. *Mol. Ther.* 17 1411–1417 10.1038/mt.2009.121 19532143 PMC2835228

[B29] Hughes M. M. Field R. H. Perry V. H. Murray C. L. Cunningham C. (2010). Microglia in the degenerating brain are capable of phagocytosis of beads, and of apoptotic cells, but do not efficiently remove PrP^Sc^, even upon LPS stimulation. *Glia* 58 2017–2030 10.1002/glia.21070 20878768 PMC3498730

[B30] Huotari J. Helenius A. (2011). Endosome maturation. *EMBO J.* 30 3481–3500 10.1038/emboj.2011.286 21878991 PMC3181477

[B31] Intra J. Salem A. K. (2008). Characterization of the transgene expression generated by branched, and linear polyethylenimine-plasmid DNA nanoparticles *in vitro*, and after intraperitoneal injection *in vivo*. *J. Control. Release* 130 129–138 10.1016/j.jconrel.2008.04.014 18538436 PMC2603176

[B32] Jiang X. S. Ni Y. Q. Liu T. J. Zhang M. Ren H. Jiang R. (2012). CCR2 overexpression promotes the efficient recruitment of retinal microglia in vitro. *Mol. Vis.* 18 2982–2992 23288990 PMC3534147

[B33] Jongstra-Bilen J. Harrison R. Grinstein S. (2003). Fcγ-receptors induce Mac-1 (CD11b/CD18) mobilization, and accumulation in the phagocytic cup for optimal phagocytosis. *J. Biol. Chem.* 278 45720–45729 10.1074/jbc.M303704200 12941957

[B34] Kawakami S. Sato A. Nishikawa M. Yamashita F. Hashida M. (2000). Mannose receptor-mediated gene transfer into macrophages using novel mannosylated cationic liposomes. *Gene Ther.* 7 292–299 10.1038/sj.gt.3301089 10694809

[B35] Kerr M. C. Teasdale R. D. (2009). Defining macropinocytosis. *Traffic* 10 364–371 10.1111/j.1600-0854.2009.00878.x 19192253

[B36] Kim B. Yang M. S. Choi D. Kim J. H. Kim H. S. Seol W. (2012). Impaired inflammatory responses in murine lrrk2-knockdown brain microglia. *PLoS ONE* 7:e34693 10.1371/journal.pone.0034693 PMC3322140 22496842

[B37] Kircheis R. Kichler A. Wallner G. Kursa M. Ogris M. Felzmann T. (1997). Coupling of cell-binding ligands to polyethylenimine for targeted gene delivery. *Gene Ther.* 4 409–418 10.1038/sj.gt.3300418 9274717

[B38] Kircheis R. Wightman L. Wagner E. (2001). Design, and gene delivery activity of modified polyethylenimines. *Adv. Drug Deliv. Rev.* 53 341–358 10.1016/S0169-409X(01)00202-2 11744176

[B39] Klegeris A. McGeer P. L. (1994). Inhibition of respiratory burst in macrophages by complement receptor blockade. *Eur. J. Pharmacol.* 260 273–277 10.1016/0014-2999(94)90351-4 7988657

[B40] Kwon E. J. Lasiene J. Jacobson B. E. Park I. K. Horner P. J. Pun S. H. (2010). Targeted nonviral delivery vehicles to neural progenitor cells in the mouse subventricular zone. *Biomaterials* 31 2417–2424 10.1016/j.biomaterials.2009.11.086 20004466 PMC2813955

[B41] Lee J. K. Chung J. McAlpine F. E. Tansey M. G. (2011). Regulator of G-protein signaling-10 negatively regulates NF-κB in microglia, and neuroprotects dopaminergic neurons in hemiparkinsonian rats. *J. Neurosci.* 31 11879–11888 10.1523/JNEUROSCI.1002-11.2011 21849548 PMC3326398

[B42] Lei L. Tzekov R. Tang S. Kaushal S. (2012). Accumulation, and autofluorescence of phagocytized rod outer segment material in macrophages, and microglial cells. *Mol. Vis.* 18 103–113 22275801 PMC3265176

[B43] Li Y. N. Qin X. J. Kuang F. Wu R. Duan X. L. Ju G. (2008). Alterations of Fc gamma receptor I, and Toll-like receptor 4 mediate the antiinflammatory actions of microglia, and astrocytes after adrenaline-induced blood-brain barrier opening in rats. *J. Neurosci. Res.* 86 3556–3565 10.1002/jnr.21810 18756515

[B44] Liu Y. Yang X. Guo C. Nie P. Ma J. (2013). Essential role of MFG-E8 for phagocytic properties of microglial cells. *PLoS ONE* 8:e55754 10.1371/journal.pone.0055754 PMC3565973 23405209

[B45] Lungwitz U. Breunig M. Blunk T. Göpferich A. (2005). Polyethylenimine-based non-viral gene delivery systems. *Eur. J. Pharm. Biopharm.* 60 247–266 10.1016/j.ejpb.2004.11.011 15939236

[B46] Lv H. Zhang S. Wang B. Cui S. Yan J. (2006). Toxicity of cationic lipids, and cationic polymers in gene delivery. *J. Control. Release* 114 100–109 10.1016/j.jconrel.2006.04.014 16831482

[B47] Maiorino C. Khorooshi R. Ruffini F. Løbner M. Bergami A. Garzetti L. (2013). Lentiviral-mediated administration of IL-25 in the CNS induces alternative activation of microglia. *Gene Ther.* 20 487–496 10.1038/gt.2012.58 22855093

[B48] Mandrekar S. Jiang Q. Lee C. Y. D. Koenigsknecht-Talboo J. Holtzman D. M. Landreth G. E. (2009). Microglia mediate the clearance of soluble Aβ through fluid phase macropinocytosis. *J. Neurosci.* 29 4252–4262 10.1523/JNEUROSCI.5572-08.2009 19339619 PMC3034143

[B49] Mantovani B. (1975). Different roles of IgG, and complement receptors in phagocytosis by polymorphonuclear leukocytes. *J. Immunol.* 115 15–17 1151058

[B50] Mantovani B. Rabinovitch M. Nussenzweig V. (1972). Phagocytosis of immune complexes by macrophages. Different roles of the macrophage receptor sites for complement (C3) and for immunoglobulin (IgG). *J. Exp. Med.* 135 780–792 10.1084/jem.135.4.780 5018051 PMC2139152

[B51] Markovic D. S. Vinnakota K. Chirasani S. Synowitz M. Raguet H. Stock K. (2009). Gliomas induce, and exploit microglial MT1-MMP expression for tumor expansion. *Proc. Natl. Acad. Sci. U.S.A.* 106 12530–12535 10.1073/pnas.0804273106 19617536 PMC2718387

[B52] Mayor S. Pagano R. E. (2007). Pathways of clathrin-independent endocytosis. *Nat. Rev. Mol. Cell Biol.* 8 603–612 10.1038/nrm2216 17609668 PMC7617177

[B53] McCoy M. K. Ruhn K. A. Martinez T. N. McAlpine F. E. Blesch A. Tansey M. G. (2008). Intranigral lentiviral delivery of dominant-negative TNF attenuates neurodegeneration, and behavioral deficits in hemiparkinsonian rats. *Mol. Ther.* 16 1572–1579 10.1038/mt.2008.146 18628756 PMC2670754

[B54] Mercer J. Helenius A. (2009). Virus entry by macropinocytosis. *Nat. Cell Biol.* 11 510–520 10.1038/ncb0509-510 19404330

[B55] Meunier A. Latremoliere A. Dominguez E. Mauborgne A. Philippe S. Hamon M. (2007). Lentiviral-mediated targeted NF-êB blockade in dorsal spinal cord glia attenuates sciatic nerve injury-induced neuropathic pain in the rat. *Mol. Ther.* 15 687–697 28192702 10.1038/sj.mt.6300107

[B56] Meunier A. Mauborgne A. Masson J. Mallet J. Pohl M. (2008). Lentiviral-mediated targeted transgene expression in dorsal spinal cord glia: tool for the study of glial cell implication in mechanisms underlying chronic pain development. *J. Neurosci. Methods* 167 148–159 10.1016/j.jneumeth.2007.07.022 17949823

[B57] Millili P. G. Selekman J. A. Blocker K. M. Johnson D. A. Naik U. P. Sullivan M. O. (2010). Structural, and functional consequences of poly(ethylene glycol) inclusion on DNA condensation for gene delivery. *Microsc. Res. Tech.* 73 866–877 10.1002/jemt.20839 20232467 PMC2930114

[B58] Milner R. Campbell I. L. (2002). The integrin family of cell adhesion molecules has multiple functions within the CNS. *J. Neurosci. Res.* 69 286–291 10.1002/jnr.10321 12125070

[B59] Mishra S. Webster P. Davis M. E. (2004). PEGylation significantly affects cellular uptake, and intracellular trafficking of non-viral gene delivery particles. *Eur. J. Cell Biol.* 83 97–111 10.1078/0171-9335-00363 15202568

[B60] Mosser D. M. Edelson P. J. (1987). The third component of complement (C3) is responsible for the intracellular survival of Leishmania major. *Nature* 327 329–331 10.1038/327329b0 3035377

[B61] Nakajima K. Hamanoue M. Shimojo M. Takei N. Kohsaka S. (1989). Characterization of microglia isolated from a primary culture of embryonic rat brain by a simplified method. *Biomed. Res.* 10 411–423

[B62] Neu M. Fischer D. Kissel T. (2005). Recent advances in rational gene transfer vector design based on poly(ethylene imine) and its derivatives. *J. Gene Med.* 7 992–1009 10.1002/jgm.773 15920783

[B63] Newland B. Aied A. Pinoncely A. V. Zheng Y. Zhao T. Zhang H. (2014). Untying a nanoscale knotted polymer structure to linear chains for efficient gene delivery in vitro, and to the brain. *Nanoscale* 6 7526–7533 10.1039/c3nr06737h 24886722

[B64] Nimmerjahn F. Ravetch J. V. (2008). Fcγ receptors as regulators of immune responses. *Nat. Rev. Immunol.* 8 34–47 10.1038/nri2206 18064051

[B65] Nitta T. Yagita H. Sato K. Okumura K. Bernstein J. J. (1992). Expression of Fcγ receptors on astroglial cell lines, and their role in the central nervous system. *Neurosurgery* 31 83–88 10.1227/00006123-199207000-00012 1386416

[B66] Ogris M. Steinlein P. Carotta S. Brunner S. Wagner E. (2001). DNA/polyethylenimine transfection particles: influence of ligands, polymer size, and PEGylation on internalization, and gene expression. *AAPS PharmSci* 3:E21 10.1208/ps030321 PMC2751016 11741272

[B67] Ogris M. Steinlein P. Kursa M. Mechtler K. Kircheis R. Wagner E. (1998). The size of DNA/transferrin-PEI complexes is an important factor for gene expression in cultured cells. *Gene Ther.* 5 1425–1433 10.1038/sj.gt.3300745 9930349

[B68] Ohsawa K. Imai Y. Kanazawa H. Sasaki Y. Kohsaka S. (2000). Involvement of Iba1 in membrane ruﬄing, and phagocytosis of macrophages/microglia. *J. Cell Sci.* 113 3073–3084 10934045 10.1242/jcs.113.17.3073

[B69] Okun E. Mattson M. P. Arumugam T. V. (2010). Involvement of Fc receptors in disorders of the central nervous system. *Neuromolecular Med.* 12 164–178 10.1007/s12017-009-8099-5 19844812 PMC2878892

[B70] Payne C. K. Jones A. S. Chen C. Zhuang X. (2007). Internalization, and trafficking of cell surface proteoglycans, and proteoglycan-binding ligands. *Traffic* 8 389–401 10.1111/j.1600-0854.2007.00540.x 17394486 PMC2715839

[B71] Pei Z. Pang H. Qian L. Yang S. Wang T. Zhang W. (2007). MAC1 mediates LPS-induced production of superoxide by microglia: the role of pattern recognition receptors in dopaminergic neurotoxicity. *Glia* 55 1362–1373 10.1002/glia.20545 17654704

[B72] Petersen H. Fechner P. M. Martin A. L. Kunath K. Stolnik S. Roberts C. J. (2002). Polyethylenimine-graft-poly(ethylene glycol) copolymers: influence of copolymer block structure on DNA complexation, and biological activities as gene delivery system. *Bioconjug. Chem.* 13 845–854 10.1021/bc025529v 12121141

[B73] Pfrieger F. W. Slezak M. (2012). Genetic approaches to study glial cells in the rodent brain. *Glia* 60 681–701 10.1002/glia.22283 22162024

[B74] Raety J. K. Lesch H. P. Wirth T. Yla-Herttuala S. (2008). Improving safety of gene therapy. *Curr. Drug Saf.* 3 46–53 10.2174/157488608783333925 18690980

[B75] Rana I. Stebbing M. Kompa A. Kelly D. J. Krum H. Badoer E. (2010). Microglia activation in the hypothalamic PVN following myocardial infarction. *Brain Res.* 1326 96–104 10.1016/j.brainres.2010.02.028 20156424

[B76] Ransohoff R. M. Cardona A. E. (2010). The myeloid cells of the central nervous system parenchyma. *Nature* 468 253–262 10.1038/nature09615 21068834

[B77] Ricklin D. Hajishengallis G. Yang K. Lambris J. D. (2010). Complement: a key system for immune surveillance, and homeostasis. *Nat. Immunol.* 11 785–797 10.1038/ni.1923 20720586 PMC2924908

[B78] Robinson A. P. White T. M. Mason D. W. (1986). Macrophage heterogeneity in the rat as delineated by two monoclonal antibodies MRC OX-41, and MRC OX-42, the latter recognizing complement receptor type 3. *Immunology* 57 239–247 3512425 PMC1453949

[B79] Ross G. D. (2000). Regulation of the adhesion versus cytotoxic functions of the Mac-1/CR3/^α^m^β^2-integrin glycoprotein. *Crit. Rev. Immunol.* 20 197–222 10.1615/CritRevImmunol.v20.i3.20 10968371

[B80] Saijo K. Glass C. K. (2011). Microglial cell origin, and phenotypes in health, and disease. *Nat. Rev. Immunol.* 11 775–787 10.1038/nri3086 22025055

[B81] Schafer D. P. Lehrman E. K. Kautzman A. G. Koyama R. Mardinly A. R. Yamasaki R. (2012). Microglia sculpt postnatal neural circuits in an activity, and complement-dependent manner. *Neuron* 74 691–705 10.1016/j.neuron.2012.03.026 22632727 PMC3528177

[B82] Sitte N. Huber M. Grune T. Ladhoff A. Doecke W. D. Von Zglinicki T. (2000). Proteasome inhibition by lipofuscin/ceroid during postmitotic aging of fibroblasts. *FASEB J.* 14 1490–1498 10.1096/fj.14.11.1490 10928983

[B83] Snyder S. L. Sobocinski P. Z. (1975). An improved 2,4,6 trinitrobenzenesulfonic acid method for the determination of amines. *Anal. Biochem.* 64 284–288 10.1016/0003-2697(75)90431-5 1137089

[B84] Sohn J. H. Bora P. S. Suk H. J. Molina H. Kaplan H. J. Bora N. S. (2003). Tolerance is dependent on complement C3 fragment iC3b binding to antigen-presenting cells. *Nat. Med.* 9 206–212 10.1038/nm814 12514742 PMC1821085

[B85] Stolzing A. Wengner A. Grune T. (2002). Degradation of oxidized extracellular proteins by microglia. *Arch. Biochem. Biophys.* 400 171–179 10.1016/S0003-9861(02)00003-6 12054427

[B86] Tang G. P. Zeng J. M. Gao S. J. Ma Y. X. Shi L. Li Y. (2003). Polyethylene glycol modified polyethylenimine for improved CNS gene transfer: effects of PEGylation extent. *Biomaterials* 24 2351–2362 10.1016/S0142-9612(03)00029-2 12699673

[B87] Thornton B. P. Vìtvièka V. Pitman M. Goldman R. C. Ross G. D. (1996). Analysis of the sugar specificity, and molecular location of the β-glucan-binding lectin site of complement receptor type 3 (CD11D/CD18). *J. Immunol.* 156 1235–1246 8558003

[B88] Ulvestad E. Williams K. Vedeler C. Antel J. Nyland H. Maerk S. (1994). Reactive microglia in multiple sclerosis lesions have an increased expression of receptors for the Fc part of IgG. *J. Neurol. Sci.* 121 125–131 10.1016/0022-510X(94)90340-9 8158203

[B89] Varkouhi A. K. Scholte M. Storm G. Haisma H. J. (2011). Endosomal escape pathways for delivery of biologicals. *J. Control. Release* 151 220–228 10.1016/j.jconrel.2010.11.004 21078351

[B90] Vedeler C. Ulvestad E. Grundt I. Conti G. Nyland H. Matre R. (1994). Fc receptor for IgG (FcR) on rat microglia. *J. Neuroimmunol.* 49 19–24 10.1016/0165-5728(94)90176-7 8294556

[B91] Větvčka V. Thornton B. P. Ross G. D. (1996). Soluble β-glucan polysaccharide binding to the lectin site of neutrophil or natural killer cell complement receptor type 3 (CD11b/CD18) generates a primed state of the receptor capable of mediating cytotoxicity of iC3b-opsonized target cells. *J. Clin. Invest.* 98 50–61 10.1172/JCI118777 8690804 PMC507400

[B92] von Gersdorff K. Sanders N. N. Vandenbroucke R. De Smedt S. C. Wagner E. Ogris M. (2006). The internalization route resulting in successful gene expression depends on both cell line, and polyethylenimine polyplex type. *Mol. Ther.* 14 745–753 10.1016/j.ymthe.2006.07.006 16979385

[B93] Ward S. A. Ransom P. A. Booth P. L. Thomas W. E. (1991). Characterization of ramified microglia in tissue culture: pinocytosis, and motility. *J. Neurosci. Res.* 29 13–28 10.1002/jnr.490290103 1886165

[B94] Wrzesinski S. Seguin R. Liu Y. Domville S. Planelles V. Massa P. (2000). HTLV type 1 tax transduction in microglial cells, and astrocytes by lentiviral vectors. *AIDS Res. Hum. Retroviruses* 16 1771–1776 10.1089/08892220050193290 11080825

[B95] Xia X. Větvička V. Yan J. Hanikýřová M. Mayadas T. Ross G. D. (1999). The β-glucan-binding lectin site of mouse CR3 (CD11b/CD18), and its function in generating a primed state of the receptor that mediates cytotoxic activation in response to iC3b-opsonized target cells. *J. Immunol.* 162 2281–2290 9973505

[B96] Xiang S. Tong H. Shi Q. Fernandes J. C. Jin T. Dai K. (2012). Uptake mechanisms of non-viral gene delivery. *J. Control. Release* 158 371–378 10.1016/j.jconrel.2011.09.093 21982904

[B97] Yue Y. Jin F. Deng R. Cai J. Dai Z. Lin M. C. M. (2011). Revisit complexation between DNA, and polyethylenimine – effect of length of free polycationic chains on gene transfection. *J. Control. Release* 152 143–151 10.1016/j.jconrel.2011.03.020 21457737

[B98] Zhang W. Dallas S. Zhang D. Guo J. P. Pang H. Wilson B. (2007). Microglial PHOX, and Mac-1 are essential to the enhanced dopaminergic neurodegeneration elicited by A30P, and A53T mutant alpha-synuclein. *Glia* 55 1178–1188 10.1002/glia.20532 17600340

